# Artificial Nanoscale Erythrocytes from Clinically Relevant Compounds for Enhancing Cancer Immunotherapy

**DOI:** 10.1007/s40820-020-00428-y

**Published:** 2020-04-13

**Authors:** Wenquan Ou, Kang Sik Nam, Dae Hoon Park, Jungho Hwang, Sae Kwang Ku, Chul Soon Yong, Jong Oh Kim, Jeong Hoon Byeon

**Affiliations:** 1 College of Pharmacy, Yeungnam University, Gyeongsan, 38541 Republic of Korea; 2 School of Mechanical Engineering, Yonsei University, Seoul, 03722 Republic of Korea; 3 College of Korean Medicine, Daegu Haany University, Gyeongsan, 38610 Republic of Korea; 4 School of Mechanical Engineering, Yeungnam University, Gyeongsan, 38541 Republic of Korea

**Keywords:** Cancer immunotherapies, Air–liquid two-phase electrospray, Paclitaxel-loaded fake blood cell Eudragit particle, Translatable chemo-immunotherapeutic nanosystems, Anti-PD-L1 antibodies

## Abstract

Because of enhanced efficacy and lower side effects, cancer immunotherapies have recently been extensively investigated in clinical trials to overcome the limitations of conventional cancer monotherapies. Although engineering attempts have been made to build nanosystems even including stimulus nanomaterials for the efficient delivery of antigens, adjuvants, or anticancer drugs to improve immunogenic cancer cell death, this requires huge R&D efforts and investment for clinically relevant findings to be approved for translation of the nanosystems. To this end, in this study, an air–liquid two-phase electrospray was developed for stable bubble pressing under a balance between mechanical and electrical parameters of the spray to continuously produce biomimetic nanosystems consisting of only clinically relevant compounds [paclitaxel-loaded fake blood cell Eudragit particle (Eu-FBCP/PTX)] to provide a conceptual leap for the timely development of translatable chemo-immunotherapeutic nanosystems. This was pursued as the efficacy of systems for delivering anticancer agents that has been mainly influenced by nanosystem shape because of its relevance to transporting behavior to organs, blood circulation, and cell–membrane interactions. The resulting Eu-FBCP/PTX nanosystems exhibiting phagocytic and micropinocytic uptake behaviors can confer better efficacy in chemo-immunotherapeutics in the absence and presence of anti-PD-L1 antibodies than similar sized PTX-loaded spherical Eu particles (Eu-s/PTX).

**Electronic supplementary material:**

The online version of this article (10.1007/s40820-020-00428-y) contains supplementary material, which is available to authorized users.

## Article Highlights

- A two-phase coaxial electrospray was designed to produce paclitaxel-loaded fake blood cell Eudragit particles (Eu-FBCP/PTX).
- The chemo-immunotherapeutic efficacy was further enhanced after combining with anti-programmed death-ligand 1 antibodies (Eu-FBCP/PTX + aPL).

**Electronic supplementary material:**

The online version of this article (10.1007/s40820-020-00428-y) contains supplementary material, which is available to authorized users.

## Graphical abstract

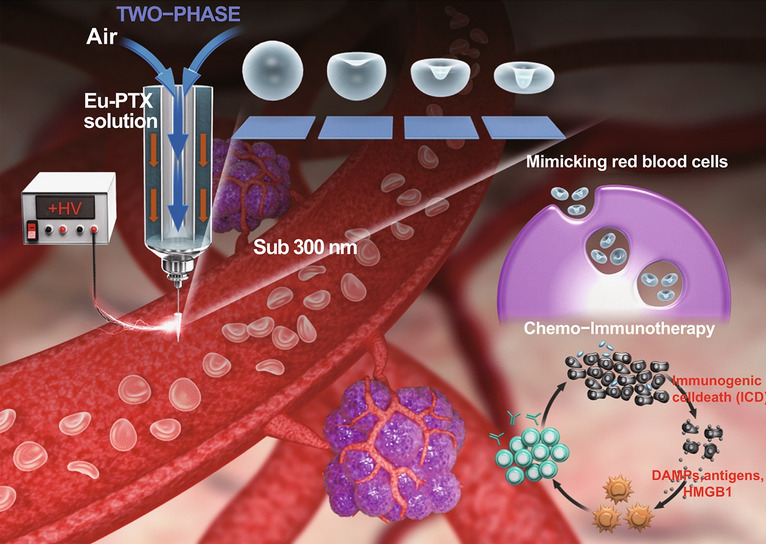

## Introduction

The recent approval of several immune-stimulatable or modulatable formulations to induce immunogenic cell death (ICD) for cancer treatments led to many attempts, including in clinical trials, to overcome the severe side effects of conventional cancer monotherapies [1–3]. The direct application of immunoreactive formulations often required a high dosage to secure immunotherapeutic efficacy; however, this approach induced unpredictable responses and adverse effects from the systemic immune activations, undesirable interactions between the formulations and host immune systems, and formulation instabilities [4]. Similar to the development of a drug delivery system (DDS), recent engineering attempts have been made to fabricate nanosystems for delivering immunoreactive formulations for the targeted release of antigens, adjuvants, or chemo-drugs to mitigate the limitations [5, 6].

The syntheses of various polymer and liposome nanoparticles have frequently been conducted as DDS to achieve the targeted delivery of immunoreactive formulations with or without chemo-drugs. More recently, inorganic nanoparticles (NPs), such as gold-, iron oxide-, silica-, carbon-, and 2D material-based NPs have also been fabricated to achieve combination cancer therapies by utilizing the abilities of the inorganic components to be stimulated by externally applied energy [7–10]. Nevertheless, these NPs are still relatively new and foreign objects, so their use is associated with biological barriers [11]. Moreover, there are challenges to be overcome regarding their safety and toxicity for practical applications [8]. There are also both high costs and technical difficulties associated with the fabrication of uniform NPs [10, 12], requiring huge efforts to ensure scalable manufacture of the NPs for timely clinical applications [13]. This background has led to a resurgence in the development of immunotherapeutic nanosystems accompanied by the use of clinically approved components, which may be a realizable option for future clinical translations into various types of tumor model [14].

**Fig. 1:**
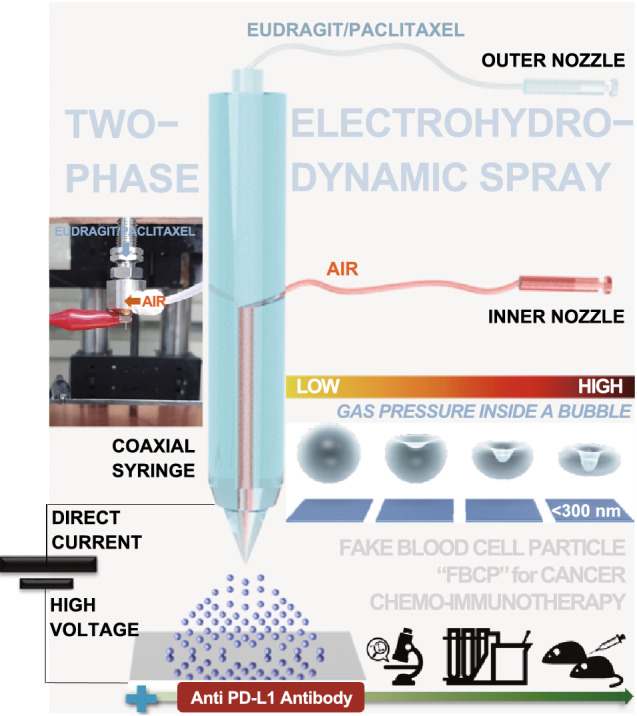
Schematic of air (inner nozzle)–liquid (outer nozzle; Eu/PTX in ethanol) two-phase electrospray (The real setup of the electrospray is shown as inset photograph.) to produce concave (mimicking the shape of blood cells) particles (Eu-FBCP/PTX) from bubble pressing of Eu/PTX melts inside drying droplets under a balance between the electrical (*B*_E_) and mechanical (*CaRe*) parameters for use in chemo-immunotherapy combined with aPL. The similarly sized spherical particles (Eu-s/PTX) were produced using a single-phase (Eu/PTX solution only) electrospray for comparison in the absence and presence of aPL

To this end, this paper presents a facile engineering method to continuously produce concave (fake blood cell) particles (FBCPs, for minimizing off-target release of the chemo-drug and immunogenicity of the nanosystem) from a clinically approved elastic polymer (Eudragit® [Eu] RS), which are used as a biomimetic nanosystem to efficiently carry anti-programmed death-ligand 1 (PD-L1) antibodies (aPL; a clinically approved immune checkpoint inhibitor) [6, 15–17]. An air–liquid two-phase electrospray is designed to fabricate Eu-FBCPs by air bubble pressing of Eu melts inside drying Eu droplets under a balance between the mechanical (capillary [Ca = *μ*_F_*V*_F_/*γ*_air–liquid_, where *μ*_F_ is the viscosity of fluid, *V*_F_ is the fluid velocity, and *γ*_air–liquid_ is the surface tension at the air–liquid interface] and Reynolds [Re = 2*ρ*_F_*r*_N_*V*_F_/*μ*_F_, where *ρ*_F_ is the density of fluid and *r*_N_ is the nozzle radius] numbers) and electrical (electrical Bond number [*B*_E_ = *ε*_F_*E*^2^*r*_N_/*γ*_air–liquid_, where *ε*_F_ is the dielectric constant of fluid, and *E* is the electric field intensity]) parameters of the spray (Fig. 1). The fabrication of FBCPs is the method selected here because mimicking erythrocytes is not only advantageous for avoiding unwanted systemic toxicity [17] but also workable for securing appropriate transporting behavior to organs, blood circulation, and cell–membrane interactions [18–21]. However, there are synthetic and instrumental challenges to overcome in order to continuously produce concave particles because of the dominance of viscous and capillary forces in the fabrication [22, 23]. This, thus, requires sacrificial discoid templates or lithographically templated substrates to fabricate concave particles, as described in previous reports, which makes the process more complex and expensive [23, 24]. To overcome the deficiencies of the template-based approaches, coaxial electrosprays adopting two different fluids have been developed for anisotropically structuring droplets during the spraying to produce concave polymers and lipid particles [25, 26]. However, this electrospray approach is not valid for fabricating NPs (only valid for microparticles) because of a short mutual diffusion distance between the small droplets [25], although smaller particles can exhibit better activities in intracellular penetration and microvascular circulation to reach distant tissues even in biomimetic delivery [27]. Although the sizes of the concave particles produced by the coaxial electrosprays are similar to those of red blood cells (6–8 μm) [24], no studies on the efficacy for anticancer applications have been performed.

The electrospray developed in this study is eventually used to produce paclitaxel (PTX; chemo-drug)-loaded Eu-FBCPs (Eu-FBCP/PTX; nanosystem to be combined with aPL as Eu-FBCP/PTX + aPL) with an average lateral dimension of about 300 nm because a combination of immune checkpoint inhibitors with chemo-drug exhibited significant enhancement of therapeutic efficacy for a broad population of patients through triggering ICD, which can improve the antitumor immunity of immune checkpoint inhibitors [28–31]. The ICD directly operates on a variety of receptors expressed by dendritic cells (DCs), promotes the presentation of tumor-associated antigen (TAA) to T cells, and stimulates a robust immune response against tumors [32]. After exposure to ICD inducers, the secretion of damage-associated molecular patterns (DAMPs) by cancer cells with the release of high mobility group box 1 (HMGB1), adenosine triphosphate (ATP) expression, and surface exposure of calreticulin (CRT) mainly contribute to the induction of ICD [31, 33]. At the pre-apoptotic stage, cancer exposed to ICD inducers not only transfers CRT to the outer leaflet plasma membrane and secretes ATP, but also releases the nuclear HMGB1 protein, which becomes permeabilized in the secondary necrosis [34]. These molecules work as stimuli to derive the recruitment of DC infiltration into the tumor microenvironment, resulting in the engulfment (stimulated by CRT) and presentation (stimulated by HMGB1) of TAA by DCs. Subsequently, the effector T cells stimulated by these processes facilitate the release of interleukin (IL)-1*β*, IL-17, and interferon (IFN)-*γ*, which act as potent agents in mediating the direct eradication of tumor cells [35].

To examine the validity of the electrospraying of clinically approved compounds, the resulting Eu-FBCP/PTX and Eu-FBCP/PTX + aPL were used in cancer chemo-immunotherapy in both in vitro and in vivo models. Compared with PTX-loaded spherical Eu particles (Eu-s/PTX), Eu-FBCP/PTX nanosystems augmented the cellular uptake into MC-38 cells through both phagocytic and micropinocytic pathways, accelerated cell cycle arrest in G2/M phase, enhanced the apoptosis of cancer cells via the intrinsic apoptotic pathway, and induced high levels of HMGB1 secretion and CRT exposure, resulting in the greater induction of ICD that triggered the maturation of DCs and activation of cluster of differentiation (CD)8^+^T cells. Because of the concave shape of Eu-FBCP/PTX, higher tumor accumulation was achieved probably due to biomimetic behavior compared with Eu-s/PTX after intravenous injection into tumor-bearing mice. Eu-FBCP/PTX + aPL further provoked DC maturation, increased CD8^+^ T cell infiltration, and activated effector T cells, resulting in a dramatic immune response against tumors. These effects therefore improved antitumor efficacy further, inhibited tumor progression, and prolonged survival time.

## Experimental

### FBCP Production

The two-phase coaxial electrospray was constructed by adopting two fluids: air (inner nozzle fluid) and Eu (EUDRAGIT® RS PO [RS, pH-independent]; Degussa, Germany)/PTX (P-9600; LC Laboratories, USA) solution (80 mg/mL, ethanol-based; outer nozzle fluid). The electrospray setup consisted of a stainless steel coaxial nozzle (NNC-DN-2230; NanoNC, South Korea), two syringe pumps (KDS200; KD Scientific Inc., South Korea), and a high-voltage direct current (DC) power supply (Ultravolt, USA), as shown in Fig. 1. The inner and outer diameters of the inner nozzle were 0.14 and 0.32 mm, respectively, and those for the outer nozzle were 0.41 and 0.70 mm, respectively. The two syringe pumps were used to precisely supply clean (particle-free) air and Eu/PTX solution into the coaxial nozzle, and the flow rates of the air and solution were set to 1 and 10 μL/min, respectively. The air was injected to produce air bubbles in electrosprayed droplets, where the bubbles pressed Eu/PTX melts during free falling of the electrosprayed droplets onto the ground plate that produced concave structures of Eu/PTX (i.e., Eu-FBCP/PTX). An electric field of 0.13 kV cm^−1^ was formed between the coaxial nozzle and ground electrode (a stainless steel plate) by applying a high DC voltage (8.5 kV) to generate high-density electric charges on the meniscus on the tip of the nozzle for electrospraying. These electrospray conditions were selected to achieve stable bubble pressing of Eu/PTX melts under a balance between the electrical and mechanical parameters. To produce Eu-s/PTX for comparison, a different electric field (0.08 kV cm^−1^) and solution concentration (10 mg mL^−1^) were applied to electrospraying. The meniscus shapes from different electrospray modes were monitored using a high-speed camera (Fastcam SA1.1; Photron, USA) with a halogen light source (KLS-100H-RS-150; Kwangwoo Co., South Korea), and the images were recorded using Motion Studio software (IDT Vision, USA).

### Physicochemical Characterization

The shapes of Eu-FBCP/PTX and Eu-s/PTX, as well as Eu-FBCP and Eu-s, were observed using a scanning electron microscope (SEM, IT-500R; JEOL, Japan) and transmission electron microscope (TEM, JEM-F200; JEOL, Japan) after the particles had been directly collected on carbon film-covered copper grids (Graphene Square, South Korea) through grid filtration using a particle sampler (Ineris, France) with a vacuum pump (3033; TSI, USA). The loadability of PTX into the Eu matrix was examined by comparison among the profiles of Eu, PTX, and Eu/PTX obtained using Raman spectroscopy (LabRam Aramis; Horiba, Japan) after samples had been deposited on flat glass disks. In-flight size distribution of Eu-FBCP/PTX was observed using a scanning mobility particle sizer (SMPS; 3936, TSI, USA).

To compare the cellular uptake between Eu-FBCP and Eu-s, cyanine 5.5 (Cy5.5) was included in Eu solution for electrospraying to form Cy5.5 containing Eu-FBCP (Eu-FBCP/Cy5.5) or Eu-s (Eu-s/Cy5.5). An analogous process was conducted for Eu RL (EUDRAGIT® RL PO [RL, pH-independent]; Degussa, Germany) to form Eu-FBCP/Cy5.5 (RL) and Eu-s/Cy5.5 (RL) for comparison of the cellular uptake. The Cy5.5 containing particles were dispersed in phosphate-buffered saline (PBS; 40 µg mL^−1^) and incubated overnight with MC-38 (Kerafast, USA) or B16/BL6 (Korean Cell Line Bank, South Korea) cells in 12-well plates (2 × 10^5^ cells/well). After 4 h, the cells were collected and washed thrice with PBS before being subjected to fluorescence-activated cell sorting (FACS; FACSCalibur, BD Biosciences, USA).

The hydrodynamic (dynamic light scattering [DLS]) size, polydispersity index (PDI), and zeta potential of Eu-FBCP/PTX and Eu-s/PTX were examined using Nano-S90 ZetaSizer (Malvern Instruments, UK) after dispersal in PBS. The shapes of dispersed Eu-FBCP/PTX in deionized water (DW), PBS, or a mixed solution (Roswell Park Memorial Institute [RPMI] + 10% fetal bovine serum [FBS; Hyclone, USA]) were further observed using TEM (H7600, Hitachi, Japan) after staining with 2% *w*/*v* phosphotungstic acid. The crystallinities of Eu-FBCP/PTX and Eu-s/PTX, as well as individual Eu and PTX for comparison were examined using an X-ray diffractometer (XRD, D/MAX-2500; Rigaku, Japan).

The stabilities in size and *EE* (from dialysis membranes [MWCO = 3500 Da; Spectrum Laboratories, USA]) of the Eu-FBCP/PTX dispersed in different media (DW, PBS, or RPMI + 10% FBS) were examined at predetermined time points (2, 4, 6, and 8 h) using the ZetaSizer and HPLC, respectively. At the end of the study, the shapes of Eu-FBCP/PTX separated from different media were further observed using the TEM after staining with 2% *w*/*v* phosphotungstic acid.

In vitro profiles of the release of PTX from Eu-FBCP/PTX at different pH (7.4 and 6.5) were obtained under shaking incubation (10 rpm min^−1^, 37 °C) in PBS. Briefly, 1 mL of Eu-FBCP/PTX suspension was sealed in a dialysis bag (MWCO = 3500 Da), and then immersed in PBS (30 mL; pH 7.4 or 6.5). At the designated time points, the amount of PTX released in the medium was measured using HPLC.

For a hemolysis assay, whole blood was withdrawn from C57BL/6 mice and centrifuged (free from plasma) at 500×*g* for 10 min. Hence, 4 *v*/*v*% erythrocyte suspension was prepared, which was incubated with an equal volume (0.5 mL) of Eu-FBCP/PTX or Eu-s/PTX suspension at different concentrations (50–400 µg mL^−1^). Erythrocytes incubated with DW (or PBS) were used as a positive (or negative) control. After shaking incubation (100 rpm min^−1^, 37 °C) for 8 h, the erythrocytes were centrifuged, and absorbance of the suspension was determined using a UV–Vis spectrophotometer (U-2800; PerkinElmer, USA) at 540 nm.

### In Vitro Cytotoxicity

Cytotoxicities of Eu-FBCP/PTX and Eu-s/PTX were examined on MC-38 cells using 3-(4,5-dimethylthiazol-2-yl)-2,5-diphenyl-tetrazolium bromide (MTT; Sigma-Aldrich, USA) assay. The cells were seeded in 96-well plates at a density of 4 × 10^3^ cells/well and incubated at 37 °C (5% CO_2_) for 24 h. These cells were then treated with Eu-FBCP/PTX and Eu-s/PTX (1–100 µg mL^−1^), as well as free PTX, Eu-FBCP, and Eu-s for comparison. After 24 h of incubation, 20 µL of MTT reagent (5.0 mg mL^−1^) was added into each well and incubated for a further 6 h before absorbance measurements using a microreader (Thermo Fisher Scientific, USA) at 570 nm.

### Cellular Uptake

MC-38 cells (1 × 10^5^ cells/well) were cultured on 12-well plates for 24 h prior to the treatments. These cells were then treated with Eu-FBCP/Cy5.5 or Eu-s/Cy5.5 (40 µg mL^−1^) for 1, 2, 4, or 6 h at 37 °C. After the incubation, the cells were harvested, washed thrice with PBS, and analyzed using a flow cytometer. Analogous procedures were conducted with different concentrations (10–80.0 µg mL^−1^) at a fixed incubation time (4 h) to analyze concentration-dependent uptake profiles.

To assess the mechanism of the uptake, MC-38 cells (1 × 10^5^ cells/well) were seeded on 12-well plates and incubated overnight. Prior to exposure to Eu-FBCP/Cy5.5 (40.0 µg mL^−1^), MC-38 cells were pretreated with 100 µM chlorpromazine hydrochloride (clathrin-mediated endocytosis inhibitor, C8138; Sigma-Aldrich), 10 mM methyl-*β*-cyclodextrin (caveolin-mediated endocytosis inhibitor, C4555; Sigma-Aldrich), or 4 µM cytochalasin D (phagocytosis and macro-pinocytosis inhibitor, PHZ1063; Invitrogen, USA) solution for 30 min at 4 °C. After 4 h, the cells were collected, washed with PBS, and analyzed using flow cytometry.

### Cell Cycle

Cell cycle and proliferation were investigated using fluorescein isothiocyanate (FITC) bromodeoxyuridine (BrdU) flow kit (Pharmingen™; BD Sciences, USA). MC-38 cells (1 × 10^5^ cells/well) were seeded on six-well plates. After 24 h of incubation, the culture medium was replaced with new cell growth medium containing 10 µM BrdU for 30 min. These cells were treated with Eu-FBCP/PTX and Eu-s/PTX, as well as PBS and free PTX (10 µg mL^−1^ PTX basis), for 12 h. The treated cells were harvested, fixed, and treated further with FITC-conjugated anti-BrdU antibodies (1:50). The cell cycle and proliferation were analyzed using flow cytometry.

### Apoptosis

Apoptosis from the treatments was analyzed using Annexin V-FITC/propidium iodide (PI) kit (BD Biosciences, USA). MC-38 cells (1 × 10^5^ cells/well) were seeded on 12-well plates and cultured for 24 h. The media were then replaced with Eu-FBCP/PTX, Eu-s/PTX, PBS, or free PTX (10 µg mL^−1^ PTX basis) and incubated for 24 h. The cells were collected, washed with PBS, stained with Annexin V-FITC/PI for 15 min, and measured using flow cytometry.

The apoptotic pathway was investigated by western blotting. Total proteins were isolated using a mammalian protein extraction reagent (M-PER™; ThermoFisher Scientific, USA) and quantified using bicinchoninic acid protein assay kit (Pierce™; ThermoFisher Scientific, USA) after centrifugation at 12,000×*g*. The treated samples (20 µg) were loaded and separated by 10% sodium dodecyl sulfate–polyacrylamide gel electrophoresis and immediately transferred to polyvinylidine difluoride membranes. Then, 5% skim milk was added to block the membranes for 1 h and incubated with primary antibodies overnight at 4 °C. Before visualization using a chemiluminescence system, the membranes were washed with Tris-buffered saline with 0.1% Tween 20 and incubated with horseradish peroxidase-conjugated secondary antibodies (1:3000) for 1 h.

To detect cytoplasmic cytochrome c (Cyt c; mouse monoclonal 1:1000, #12,963; Cell Signaling Technology, USA), Cyt c released into the cytosol was observed using a mitochondrion/cytosol fractionation kit (ab65320; Abcam, UK).

To assess mitochondrial damage, MC-38 cells (1 × 10^5^ cells/well) were treated with Eu-FBCP/PTX and Eu-s/PTX, as well as PBS and free PTX (10 µg mL^−1^ PTX basis) for 24 h. The cells were washed thrice with PBS and stained with JC-1 fluorescent dye (10 µg mL^−1^, T3168; Invitrogen, USA) for 30 min at 37 °C. The mitochondrial damage was analyzed using flow cytometry.

The apoptosis was further examined using live/dead cell assay through acridine orange (AO)/PI staining. MC-38 cells (1 × 10^5^ cells/well) were cultured on 12-well plates and incubated overnight. The cells were then treated with Eu-FBCP/PTX and Eu-s/PTX, as well as PBS and free PTX (10 µg mL^−1^ PTX basis) for 24 h. The treated media were replaced with AO (6.7 μM, Invitrogen, USA) and PI (750 μM, Sigma-Aldrich, USA) dissolved in Dulbecco’s modified Eagle’s medium (Hyclone, USA) and incubated for 20 min. After PBS washing, the cells were finally visualized using a fluorescence microscope (Eclipse Ti; Nikon Instruments, USA).

### Maturation of Bone Marrow-Derived Dendritic Cells (BMDCs) and Activation of CD8^+^ T Cells

The release of DAMP molecules, CRT (rabbit monoclonal 1:1000, #12238; Cell Signaling Technology, USA), and HMGB1 (rabbit monoclonal 1:1000, #6893; Cell Signaling Technology, USA) from tumor cells treated with Eu-FBCP/PTX and Eu-s/PTX was investigated through western blotting. For BMDC maturation analysis, bone marrow cells were harvested from the femur of C57BL/6 mice and stimulated for 7 days in the presence of recombinant murine granulocyte–macrophage colony-stimulating factor (20 ng mL^−1^, PeproTech, USA) and IL-4 (10 ng mL^−1^, PeproTech, USA). The generated immature BMDCs (4 × 10^4^ cells/well) were seeded in a 96-well plate (U bottom) and co-cultured with MC-38 cells that had been pretreated with Eu-FBCP/PTX and Eu-s/PTX, as well as PBS and free PTX (10 µg mL^−1^ PTX basis), for 24 h. After 16 h of treatment, the cells were harvested and stained with PE-anti-mouse CD11c, PerCP/Cy5.5 anti-mouse I-A/I-E, and FITC-anti-mouse CD86 (BioLegend, USA) for 15 min for flow cytometry.

The activation of CD8^+^ T cells (isolated using mouse CD8^+^ T cell isolation kit [Miltenyi Biotec, Germany]) was investigated using carboxyfluorescein succinimidyl ester (CFSE) cell proliferation kit (CellTrace™; Invitrogen, USA). CD8^+^ T cells (1 × 10^6^ cells/well) were labeled with 5 µM CFSE and seeded on 96-well plates coated with 5.0 µg mL^−1^ anti-CD3 Ab (Biolegend, USA). The pretreated BMDCs were then added into each well and incubated for 48 h. The cells were stained with PE anti-mouse CD3, PerCP/Cy5.5 anti-mouse CD8, and adenomatous polyposis coli protein anti-mouse IFN-*γ* antibodies (BioLegend, USA) and analyzed using flow cytometry (FACSVerse; BD Biosciences, USA).

### In Vivo Imaging

To investigate biodistribution, the fluorescence generated from the Cy5.5-labeled Eu-FBCP or Eu-s at different concentrations (1.5625–50 µg mL^−1^) was observed using an in vivo animal imaging system (FOBI; NeoScience, South Korea). MC-38 cells (1 × 10^6^ cells/mouse) were subcutaneously administered into the flank of C57BL/6 mice to generate MC-38 tumors (> 200 mm^3^). The Eu-FBCP/Cy5.5 and Eu-s/Cy5.5, as well as free Cy5.5 (1.5 mg kg^−1^ Cy5.5 basis) for comparison, were then intravenously injected into the mice. The fluorescence distribution in the treated mice (hearts, livers, spleens, lungs, kidneys, and tumors) was obtained at 0, 4, 8, 12, and 24 h using the imaging system after the mice had been sacrificed.

### In Vivo Therapeutic Study

In vivo MC-38 tumor models were constructed by the subcutaneous administration of MC-38 cells (1 × 10^6^ cells/mouse) into the flank of C57BL/6 mice. When tumor size reached ~ 100 mm^3^, the mice were randomly divided into seven treatment groups (six mice per group): (1) PBS, (2) free PTX, (3) Eu-s/PTX, (4) Eu-FBCP/PTX, (5) aPL, (6) Eu-s/PTX + aPL, and (7) Eu-FBCP/PTX + aPL. The mice were treated via intravenous injection with these different configurations (1–7; 5.0 mg/kg PTX and 1.5 mg kg^−1^ aPL basis) every 3 days until the end of the study. During the period, the tumor volume (width^2^ × length × 0.5) and body weight were recorded every 2 days. To analyze the antitumor immune response, tumors from the treated mice were isolated and dissociated into single-cell suspensions by mechanical passage through 100-µm strainers (Falcon; BD Biosciences, USA). The tumor-infiltrating immune cells were then enriched using Ficoll-Paque density gradient media (GE Healthcare Life Sciences, USA) and stained with fluorescence-labeled anti-mouse CD45, CD3, CD11c, CD86, MHC II, CD4, CD8, and Foxp3 antibodies. Intratumoral DCs, CD4, and CD8 were identified using flow cytometry (FACSVerse; BD Biosciences, USA). Levels of the tumor necrosis factor-*α* (TNF-*α*) in the collected homogenate were also analyzed after centrifugation using enzyme-linked immunosorbent assay kit (Biolegend, USA). The analysis of intracellular IFN-*γ* and granzyme B secreted by CD8^+^ T cells was conducted through stimulation of the isolated immune cells with tumor antigen, phorbol 12-myristate 13-acetate (50 ng mL^−1^; Sigma-Aldrich), ionomycin (500 ng mL^−1^; Sigma-Aldrich), and GolgiStop (1 μL mL^−1^; BD Biosciences, USA) for 4 h. The analysis was subsequently performed using flow cytometry (FACSVerse; BD Biosciences, USA) after staining with fluorescence-labeled anti-mouse CD45, CD3, CD4, CD8, IFN-*γ*, and granzyme B antibodies (BioLegend, USA). The heart, liver, lungs, kidneys, and tumors from the treated mice were finally dissociated, weighed, and stained with hematoxylin and eosin (H&E). The expression levels of CD31, Ki-67, cleaved caspase-3 (rabbit monoclonal 1:1000, #9664; Cell Signaling Technology, USA), cleaved caspase-9 (rabbit monoclonal 1:1000, #20750; Cell Signaling Technology, USA), HMGB1, CRT, and CD8 in the tumor sections were further examined through immunohistochemical assays.

To establish immunocompromized mouse models, including CD4-deficient, CD8-deficient, and CD4–CD8-deficient mice, C57BL/6 mice were intraperitoneally injected with mouse IgG (5.0 mg kg^−1^; SouthernBiotech, USA), anti-CD4 (5.0 mg kg^−1^, GK1.5; BioXcell, USA), anti-CD8 (5.0 mg kg^−1^, 2.43; BioXcell, USA), and anti-CD4^+^ anti-CD8 antibodies. When the tumor size reached ~ 100 mm^3^, the mice were intravenously injected with Eu-FBCP/PTX or Eu-FBCP/PTX + aPL, and the tumor volume and body weight were monitored during the treatment period.

All mouse experiments were approved and carried out in accordance with the instructions and guidelines of the Institutional Animal Ethics Committee, Yeungnam University, Republic of Korea.

### Statistical Analysis

The analyses were conducted using commercial software (Prism 7.0; GraphPad Software, USA). All experimental results are presented as the mean ± standard deviation. One-way analysis of variance (for more than two groups) and Student’s *t* test (for pairs of groups) were used to analyze the statistical significance of differences. Log-rank test was used for statistical analysis of in vivo survival data. The statistical significance is indicated as *p < 0.05 and **p < 0.01.

## Results and Discussion

### Preparation and Characterization

**Fig. 2:**
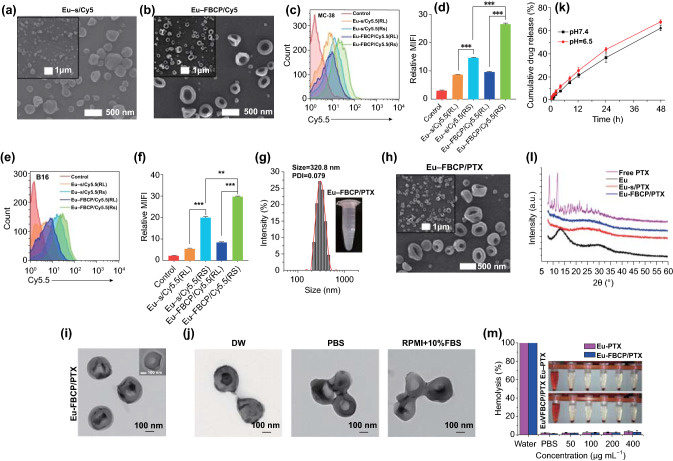
Characterization of Eu-FBCP and Eu-s in the absence and presence of PTX. **a**,** b** High- and low-magnification SEM images of Eu-s/Cy5.5 and Eu-FBCP/Cy5.5 from electrosprays of Cy5.5 containing Eu liquid. Similarly sized Eu-s/Cy5.5 was prepared by controlling the concentration of Eu dissolved in ethanol in the absence of air injection into the inner nozzle. **c**–**f** FACS results (*n* = 3; fluorescence profile and mean fluorescence intensity [MFI]) for comparing cellular uptake in MC-38 (**c**,** d**) or B16 (**e**,** f**) cells between Eu-FBCP and Eu-s after adding Cy5.5 to the electrosprays to examine the greater uptake of Eu-FBCP because of the concave shape. This assay included Eu RL to confirm the differences in uptake between the Eu RS and Eu RL for justification of the selection of Eu RS. **g** DLS size distribution of Eu-FBCP/PTX dispersed in PBS (inset digital image) exhibiting mean size and PDI. **h**,** i** High- and low-magnification SEM (**h**) and TEM (**i**) images of Eu-FBCP/PTX. **j** Representative TEM images of Eu-FBCP/PTX after 8 h dispersion in DW, PBS, or RPMI + 10% FBS to examine the hydrodynamic stabilities for different media. **k** In vitro release profiles of PTX from Eu-FBCP/PTX for 48 h dispersion at pH 6.5 or pH 7.4 (*n* = 3). **l** XRD spectra of Eu-FBCP/PTX and Eu-s/PTX, as well as free PTX and Eu (RS; before the electrosprays), to examine and compare incorporation between Eu and PTX. **m** Hemolysis results from 8 h incubation of red blood cells with Eu-FBCP/PTX or Eu-s/PTX at different concentrations (50–400 µg mL^−1^) (*n* = 3; **p < 0.01 and **p < 0.001). Insets show representative digital images of the incubated dispersions

Air bubble pressing was selected to generate the concave structure of Eu-FBCP/PTX because the exponential increase of bubble pressure inside drying droplets can be induced by decreasing their size [36], which compresses Eu/PTX melts to build FBCP. To ensure stable conditions for bubble pressing, the characteristic balance ($B_{c} = \left[ {\frac{{B_{E}^{2} }}{\text{CaRe}}} \right]^{1/3}$, where Ca, Re, and *B*_E_ are Ca_2_/Ca_1_, Re_2_/Re_1_, and *B*_E,2_/*B*_E,1_, and subscripts 1 and 2 represent Eu/PTX liquid and air, respectively) [37] was analyzed by modulating the ratio of volumetric flow rate between *Q*_1_ (Eu/PTX liquid) and *Q*_2_ (air), as shown in Fig. S1. The concave structure was formed in the *Q*_2_/*Q*_1_ range of 0.1–1.0 (i.e., plateau region of the parameters), and 0.1 of *Q*_2_/*Q*_1_ was selected in this study to secure an ideal balance (*B*_c_ ~ 1). This value was valid to scale the bubble diameter (*d*_B_) for pressing Eu/PTX melts to generate the concave structure using Eq. 1 [38]:

1 $$d_{{\text{B}}} = \frac{{cP_{{\text{B}}} }}{{Q_{1} \mu_{1} }}$$

where *c* and *P*_B_ are the experimental constant and bubble pressure, respectively. *P*_B_ can be predicted using Eq. 2 [36]:

2 $$P_{{\text{B}}} = P_{0} + kv_{{\text{B}}} + \gamma_{{\text{air}{-}\text{ liquid}}} /d_{{\text{B}}}$$

where *P*_0_ is the surrounding pressure, and *k* and *v*_B_ are the compression modulus and proportion of a droplet occupied by bubbles, respectively. Simultaneously solving these two equations having both *d*_B_ and *P*_B_ resulted in an appropriate *μ*_1_ (2.4 mPa s; viscosity of Eu/PTX liquid) for securing electrospray cone-jet breakup to stably produce Eu-FBCP/PTX nanosystems. Figure S1 also shows the meniscus shape of the selected operating conditions, where a cone-jet was formed to spray Eu/PTX liquid containing air. To compare cellular uptake between Eu-FBCP and Eu-s, cyanine 5.5 (Cy5.5)-supplemented Eu solution was used for the electrospray to fabricate Cy5.5-labeled Eu-FBCP (Eu-FBCP/Cy5.5) or Eu-s (Eu-s/Cy5.5); the morphologies of the resulting particles were observed using a SEM, as shown in Fig. 2a, b. The designed two-phase electrospray successfully produced concave structures, which implies that the bubble pressing of drying Eu solutes is workable to overcome viscous force and surface tension (preferable to form spherical shapes) for generating anisotropic shapes. These Cy5.5-labeled nanosystems were exposed to MC-38 and B16BL/6 tumor cells to examine the differences in cellular uptake between the Eu-FBCP/Cy5.5 and Eu-s/Cy5.5 (Fig. 2c–f). To justify the use of Eu (model RS in this study), analogous Cy5.5-labeled nanosystems (Eu-FBCP/Cy5.5 [RL] and Eu-s/Cy5.5 [RL]) were also fabricated by replacing Eu with Eu (RL) for the preparation of Eu/Cy5.5 solution. Dynamic light scattering (DLS) size distributions of the four configurations are summarized in Table S1. There were no significant differences between the configurations (about 320 nm), which suggests extendability of the developed electrospray for other analogous substances. The FACS profiles show that Cy5.5-labeled nanosystems from Eu (RL) exhibited significantly lower cellular uptake (less than half) in both MC-38 and B16BL/6 cells than those from Eu-FBCP/Cy5.5 or Eu-s/Cy5.5 (Fig. 2c–f). These differences in uptake may result from the different fractions of functional quaternary ammonium groups between the Eu and Eu (RL) [39]. To examine PTX incorporation with Eu, the resulting powder form of Eu-FBCP/PTX (Fig. S2A) was subjected to Raman spectroscopy to compare its spectrum with those from individual Eu-FBCP and PTX (Fig. S2B). The attenuated band intensities at around 610, 1050, 1720, and 3000 cm^−1^ attributed to C=C–C, C–O, C=O, and C–H groups in PTX, respectively, were observed for Eu-FBCP/PTX compared with the free PTX [40], demonstrating the distribution of PTX inside the Eu matrix. The DLS distribution of Eu-FBCP/PTX exhibited a mean size of 320.8 nm with PDI of 0.079 (Fig. 2g), which matched the size distributions observed using electron microscopes (Fig. 2h, i). In-flight size distribution of Eu-FBCP/PTX right after electrospraying was measured through direct vacuum sampling using a SMPS that exhibits median diameter (Fig. S2C) of 328 nm, corresponding to other size distribution results. From the microscopic observations, the shapes of Eu-FBCP/PTX were identified as concave structures even with the inclusion of PTX, suggesting that the developed electrospray is also workable for a mixture liquid to form concave structures. In the absence of air injection, spherical particles (i.e., Eu-s/PTX) were produced instead of the concave shape (Fig. S2A), proving the critical role of air injection. In addition, black phosphorus (BP) NPs were included in the Eu/PTX solution for the electrospray, and a representative TEM image and its elemental maps were observed (Fig. S2D). The BP NPs (green dots) were distributed in the concave particle, which implies that harder NPs can also be included in FBCP without the use of pre- or posttreatment. For comparison in biological assays, similarly sized Eu-s/PTX nanosystems were fabricated from a single-phase electrospray, and their size distribution and shape were confirmed using DLS and SEM-TEM measurements after dispersion (Fig. S3). In particular, no contrast gradients were found in the central region of Eu-s/PTX in the TEM observation, proving the relatively rigid core property of Eu-s/PTX. The loading capacity (LC) and encapsulation efficiency (EE) of Eu-FBCP were 14.6% and 86.7%, respectively, exhibiting high loading capacities of PTX (Fig. S4). Stabilities in the size distribution and *EE* were examined for 8 h dispersion in DW, PBS, or RPMI + 10% FBS, exhibiting no significant changes in the size and *EE* (loss of PTX content) (Fig. S5). The size distributions at 8 h still showed mean sizes of about 300 nm (Fig. S6), although there were minor deformations (maintaining concave structures), probably due to hydrolysis during dispersion in the media (Fig. 2j). Eu-FBCP/PTX nanosystems demonstrated time-dependent sustained release profiles (matched HIGUCHI model) for 48 h dispersion (Fig. 2k). There were no drastic increases in PTX release at pH 6.5 (mimicking the tumor microenvironment) compared with that under physiological conditions (pH 7.4), and about 60% of PTX was released after 48 h dispersion, suggesting that the nanosystems may be suitable for targeted sustained PTX release during long-term blood circulation because of the stable concave structure. The morphological stability and sustained release may occur because of the successuful incorporation of PTX into the Eu matrix during the fabrication, which were further examined using XRD (Fig. 2l). The profiles of both Eu-FBCP/PTX and Eu-s/PTX revealed distinct differences compared with those of individual PTX and Eu, proving the uniform distribution of PTX in the vicinity of Eu molecules (showing broadened profiles). In the hemolysis assay (Fig. 2m), no notable hemolytic effects were observed from 8 h of treatment with Eu-FBCP/PTX or Eu-s/PTX nanosystems even at high concentrations (> 200 µg mL^−1^) compared with the PBS-treated control group, suggesting hemocompatibility for safe systemic circulation of the nanosystems.

### In Vitro Cell Viability and Cellular Uptake

**Fig. 3:**
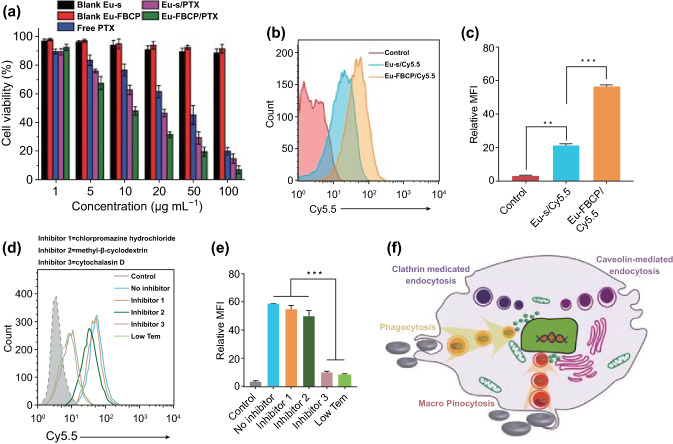
In vitro bioassay results and plausible mechanism after MC-38 cells were treated with Eu-FBCP/PTX (**p < 0.01 and **p < 0.001). **a** Cytotoxicities of MC-38 cells treated with Eu-FBCP/PTX or Eu-s/PTX for 24 h, as well as free PTX and individual Eu-FBCP or Eu-s (*n* = 6). **b**,** c** FACS results (*n* = 3; fluorescence profile and MFI) for comparing cellular uptake between Eu-FBCP/Cy5.5 and Eu-s/Cy5.5. **d**,** e** FACS results (*n* = 3; fluorescence profile and MFI) for comparing cellular uptake of Eu-FBCP/Cy5.5 between the pretreatments with different inhibitors, including low-temperature conditions. **f** Schematic of a plausible mechanism for the uptake pathway from the pretreatment of cells with the different inhibitors and subsequent incubation with Eu-FBCP/Cy5.5

Based on these physicochemical properties, the in vitro anticancer efficacy of Eu-FBCP/PTX nanosystems, as well as Eu-s/PTX, Eu-FBCP, Eu-s, and free PTX for comparison was explored in MC-38 (murine colon adenocarcinoma) cells. Both Eu-FBCP and Eu-s (in the absence of PTX) exhibited high cell viability (> 95%), as revealed by MTT assays (Fig. 3a), whereas free PTX induced dose-dependent cytotoxicity, showing a high half-maximal inhibitory concentration (IC_50_) of 28.4 µg mL^−1^. The IC_50_ value was significantly reduced (15.9 µg mL^−1^; nearly half that from free PTX), probably because of the effect of nano-drug delivery when PTX was loaded into Eu-s, and the value was further reduced (9.7 µg mL^−1^; near one-third) by a synergistic effect of the nano-size and concave shape (deriving preferential behaviors in cellular uptake) when PTX was loaded into Eu-FBCP. To confirm the further enhancement in cancer cell killing, FACS analyses were performed for comparison between Cy5.5-labeled Eu-FBCP and Eu-s in both time- (Fig. S7A–D) and dose-dependent configurations (Fig. S7E–H) in MC-38 cells. The results led to the conclusion that Eu-FBCP/Cy5.5 potentiates threefold more cellular uptake than Eu-s/Cy5.5 (Fig. 3b, c) by providing more contact surfaces to interact with cell surfaces [41], contributing to the highest activity of cancer cell killing. The mechanism of uptake of Eu-FBCP/Cy5.5 was specifically identified by using three chemical inhibitors, chlorpromazine (inhibitor 1; clathrin-mediated endocytosis inhibitor), methyl-*β*-cyclodextrin (inhibitor 2; caveolin-mediated endocytosis inhibitor), and cytochalasin D (inhibitor 3; phagocytic and macropinocytosis inhibitor), as shown in Fig. 3d, e [42, 43]. Compared with the case without inhibitor treatment, the treatments with inhibitors 1 and 2 did not induce notable changes in Eu-FBCP/Cy5.5 uptake, whereas the treatments with inhibitor 3 and low-temperature (Low temp.)-mediated significant reductions in the uptake. These results indicate that Eu-FBCP/Cy5.5 entered the cells mainly through phagocytic and micropinocytic pathways, probably because of their concave nature, as depicted in Fig. 3f, although the uptake may also be caused by endosomal/lysosomal degradation. Nevertheless, this may provide in vivo potential not only to make contact with macrophages difficult but also to achieve long-term circulation in blood vessels [44, 45].

### Cell Cycle and Apoptosis

**Fig. 4:**
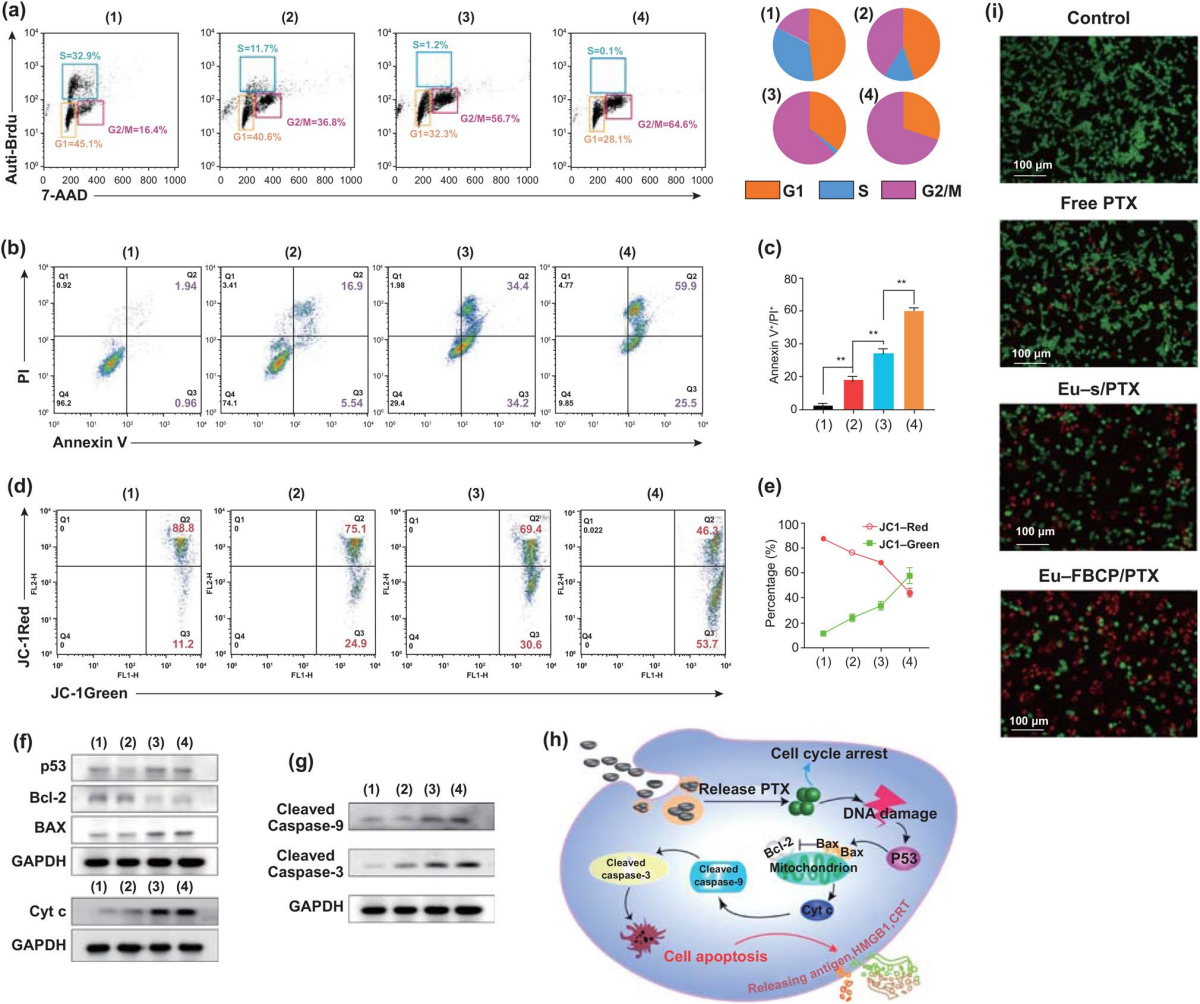
In vitro cell cycle and apoptotic analyses for MC-38 cells treated with different configurations (1: control, 2: free PTX, 3: Eu-s/PTX, and 4: Eu-FBCP/PTX). **a** Cell cycle analysis results from the different treatments (1–4). Brdu and PI were used to calculate the cell fractions for each phase (G1, S, or G2/M) in the cell cycle. **b**,** c** Apoptotic profiles and the quantified data from the 24 h treatments (1–4) using Annexin V-FITC/PI kit (*n* = 3). **d**,** e** FACS profiles and the quantified data from JC-1 staining exhibiting changes in mitochondrial membrane potential for the 24 h-treated (1–4) cells (*n* = 3). **f**,** g** Representative expression of p53, Bcl-2, Bax, Cyt c, cleaved caspase-9, and cleaved caspase-3 in the treated cells (1–4). **h** Schematic of a plausible mechanism for the apoptosis in the treated cells (1–4) based on the expression (**f**,** g**). **i** Representative microscopic AO/PI-stained cell images from live/dead assay with the treatments (1–4)

In vitro cell cycle analysis using BrdU and PI staining revealed that free PTX treatment (case 2) significantly reduced the proliferation of MC-38 cells (representing S phase) to 11.7% compared with that in the control group (case 1; 32.9%) (Fig. 4a). Loading PTX into Eu-s (case 3; Eu-s/PTX) further decreased the fraction of S phase down to 1.2% while increasing the fraction of G2/M phase to 56.7%. The fractions of S and G2/M for Eu-FBCP/PTX (case 4) were 0.1% and 64.6%, respectively, indicating that more cells had entered the apoptotic pathway. In other words, Eu-FBCP/PTX provoked apoptosis in 85.4% of cells (early apoptosis and late apoptosis: Q2 + Q3), exhibiting 16.8% more apoptosis than those from Eu-s/PTX (Fig. 4b, c). The loss of mitochondrial transmembrane potential (Δ*Ψ*_m_) was also monitored using JC-1 staining. Driven by the Δ*Ψ*_m_, JC-1 was concentrated in mitochondria and generated red-emitting aggregates in the mitochondria, rather than in the cytosol (presenting green fluorescence). The variation of red/green JC-1 fluorescence can thus be harnessed to measure the Δ*Ψ*_m_ [46, 47], and the disruption in Δ*Ψ*_m_ has been indicated as an important hallmark of Cyt c translocation from mitochondria to cytosol; thus, it can be considered as an initiator of the apoptotic process [48]. As shown in Fig. 4d, e, cells treated with free PTX (case 2) exhibited reduced red fluorescence (75.1%) compared with untreated cells as a control (case 1; 88.8%), but increased green fluorescence (24.9%). For the treatment with Eu-FBCP/PTX, this inverse proportion eventually reached 46.5% and 53.7% red and green fluorescence, respectively, suggesting that the greatest disruption in Δ*Ψ*_m_ (mitochondrial damage) of the cancer cells can be driven by Eu-FBCP/PTX treatment. Inspired by the enhanced apoptosis and mitochondrial damage, the proteins related to the intrinsic apoptotic pathway were also explored by western blotting. After the treatment with Eu-FBCP/PTX (case 4), the expression of apoptotic p53 and Bax proteins was intensified, while the levels of the anti-apoptotic protein Bcl-2 were significantly reduced, leading to the release of more Cyt c into the cytosol and the upregulation of cleaved caspase-9 and -3 apoptotic proteins (Fig. 4f, g). In this apoptotic pathway, the DNA damage caused by PTX released from Eu-FBCP/PTX triggered p53 that can directly activate Bax and neutralize Bcl-2 at the mitochondria, resulting in the release of Cyt c from mitochondria (Fig. 4h). The upregulated Cyt c then induced high levels of cleaved caspase-9, and subsequently elevated cleaved caspase-3 expression, triggering apoptosis [49, 50]. Although similar trends were observed in the cells treated with free PTX and Eu-s/PTX, their efficacies were significantly lower than those from Eu-FBCP/PTX, representing the synergistic apoptotic effect of the nano-size and concave shape. Consequently, the live/dead cell assay using AO/PI staining exhibited the greatest proportion of apoptotic cells after treatment with Eu-FBCP/PTX, reflecting the strongest effect of cancer cell killing (Fig. 4i).

### Immunogenic Cell Death-Induced Immune Response

**Fig. 5:**
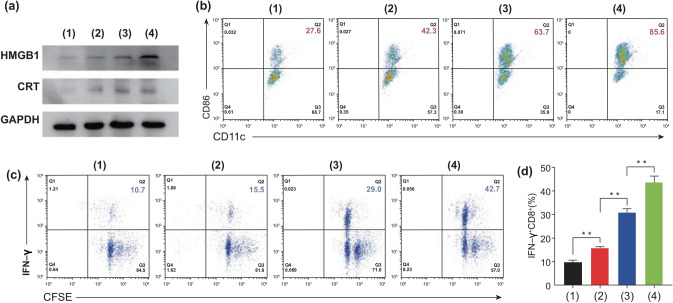
In vitro bioassays for MC-38 cells to examine ICD-induced DC maturation and CD8^+^ T cell activation from different treatments (1: control, 2: free PTX, 3: Eu-s/PTX, and 4: Eu-FBCP/PTX). **a** Representative expression of HMGB1 and CRT from the treatments (1–4), indicating the induction of ICD. **b** CD11c^+^–CD86^+^ (as indicators of DC maturation) plots to identify maturation of the DCs by exposure to TAA generated from the treated cells (1–4). **c** CFSE–IFN-*γ* plots to determine the proliferation and activation of CD8^+^ T cells. **d** Fractions of activated IFN-*γ*^+^CD8^+^ T cells from the treatments (1–4) (*n* = 3; **p < 0.01)

An anticancer chemo-drug, PTX, including doxorubicin and cyclophosphamide, is a comparable option to photodynamic and radiation therapies for inducing ICD via the release of the tumor antigens DAMPs, such as HMGB1, CRT, and ATP, from the surface of dying cells [51, 52]. These can provide adjuvants to stimulate DCs and maintain vital roles in initiating cancer immunotherapy [32, 53]. In particular, the secretion of HGMB1 and CRT was demonstrated to be a key factor for provoking the maturation of DCs to initiate ICD [54]. In the western blot analysis (Fig. 5a), significantly higher expression of HMGB1 and CRT was observed in the cells treated with Eu-FBCP/PTX compared with that from Eu-s/PTX or free PTX. The presence of these molecules as adjuvant stimuli for DC maturation induced 85.6% mature DCs, which was 21.9% more than that from Eu-s/PTX (63.7%) and more than double that from free PTX (42.3%) (Figs. 5b, S8). The mature DCs could present TAAs and prompt the proliferation, differentiation, and activation of T cells, leading to the initiation of anticancer immune responses [52, 55]. In this regard, in the Eu-FBCP/PTX-treated group, the substantial proliferation and activation of CD8^+^ T cells were activated after co-culturing the TAA-captured DCs with CD8^+^ T cells, generating 42.7% CD8^+^ T cells (the highest level compared with the treatments with Eu-s/PTX and free PTX), which proliferated and were activated (Fig. 5c, d). These results revealed that Eu-FBCP/PTX treatment is most workable to maximize CRT and HMGB1 expression, enhancing the maturation of DCs and activation of anticancer IFN-*γ*^+^CD8^+^ T cells.

### In Vivo Biodistribution

**Fig. 6:**
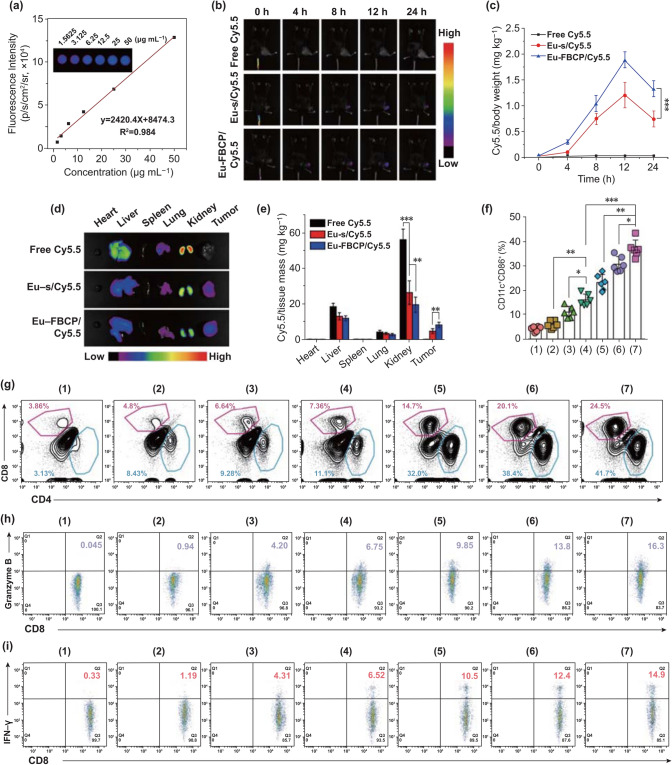
In vivo biodistributions of Eu-FBCP/Cy5.5 (or Eu-s/Cy5.5) and antitumor immune responses from different treatments (1: PBS, 2: free PTX, 3: Eu-s/PTX, 4: Eu-FBCP/PTX, 5: aPL, 6: Eu-s/PTX + aPL, and 7: Eu-FBCP/PTX + aPL). **a** Calibration curves of fluorescent Cy5.5 in the concentration range of 1.5625 to 50.0000 µg/mL obtained using an animal imaging system after in vivo exposure. **b** Time profiles (0, 4, 8, 12, and 24 h) of the fluorescence to examine the tumor accumulation of Eu-FBCP/Cy5.5 and Eu-s/Cy5.5, as well as free Cy5.5 for comparison after intravenous injection. **c** Cy5.5 distributions in tumors for the time points. **d**,** e** Representative fluorescence images and the quantified data for major organs and tumors collected from the 24 h-treated mice. **f** Intratumoral maturation of the DCs from intravenous injections with different treatments (1–7) (*n* = 6; *p < 0.05, **p < 0.01, and ***p < 0.001). **g** Plots of CD4^+^–CD8^+^ T cells infiltrated into the tumor microenvironment after the treatments (1–7). **h**,** i** Plots of CD8^+^–granzyme B^+^ and CD8^+^–IFN-*γ*^+^ levels from intratumorally infiltrated CD8^+^ T cells after the treatments (1–7)

In vivo tumor accumulation was investigated using Cy5.5-labeled Eu-FBCP (Eu-FBCP/Cy5.5) nanosystems, as well as Eu-s and free Cy5.5 for comparison. Before applying the nanosystems to in vivo models, the linearity of fluorescence intensity upon increasing the dose was examined and identified well, indicating the suitability for quantifying the amount of Cy5.5 in vivo (Fig. 6a). Fluorescence images of the mice with intravenous injection of Cy5.5-labeled nanosystems were captured to analyze the accumulation profiles (fluorescence contours) of the nanoystems over time (0–24 h). The fluorescence in tumor regions generated after the injection of Eu-FBCP/Cy5.5 or Eu-s/Cy5.5 gradually increased until 12 h, and revealed decreased intensities at 24 h (Fig. 6b, c). Eu-FBCP/Cy5.5-treated mice exhibited significantly higher (1.76-fold) fluorescent intensity in tumor regions than those from Eu-s/Cy5.5 treatment, while no observable fluorescence was found in the mice treated with free Cy5.5. According to the fluorescent distributions in major organs collected from the treated mice, the loading of Cy5.5 into Eu-FBCP or Eu-s significantly reduced the Cy5.5 accumulation in liver, kidneys, and lungs, enhancing the tumor-selective accumulation of Cy5.5 compared with that upon free Cy5.5 treatment (Fig. 6d, e). The highest tumor accumulation of Eu-FBCP/Cy5.5 nanosystems proved again their enhanced circulation and cellular uptake compared with those of Eu-s/Cy5.5.

### In Vivo Antitumor Immunotherapy

To confirm the crucial role of Eu-FBCP/PTX nanoystems for ICD-induced immunotherapy, relevant in vivo models were further constructed to examine the maturation of DCs and activation of antitumor IFN-γ^+^CD8^+^ T cells in the absence and presence of aPL. To perform this, MC-38 tumor-bearing mice were treated with Eu-FBCP/PTX (case 4) or Eu-FBCP/PTX + aPL (case 7), as well as Eu-s/PTX (case 3), Eu-s/PTX + aPL (case 6), free PTX (case 2), and aPL (case 5) for comparison. In the absence of aPL, Eu-FBCP/PTX nanosystems could stimulate substantial DC maturation (CD11c^+^CD86^+^) in tumor sites, exhibiting 2.1-fold higher levels than free PTX and 1.3-fold higher than Eu-s/PTX (Fig. 6f). In the presence of aPL, 2.4- or 1.6-fold more mature DCs were identified from the treatment with Eu-FBCP/PTX + aPL compared with those upon the treatments with Eu-FBCP/PTX or free aPL. Eu-FBCP/PTX + aPL further induced a high percentage of CD8^+^ T cells (24.5%) infiltrating into the tumor microenvironment, which was 4.4% higher than for Eu-s/PTX + aPL (20.1%), 3.3-fold higher than for Eu-FBCP/PTX (7.36%), and 1.6-fold more than for aPL (14.7%) (Figs. 6g, S9A). Meanwhile, Eu-FBCP/PTX + aPL substantially elevated the percentage of tumor-infiltrating granzyme B^+^CD8^+^ activated T cells to 16.3%, exhibiting greater activity than their counterparts Eu-s/PTX + aPL (13.8%), Eu-FBCP/PTX (6.75%), and aPL (9.85%) (Figs. 6h, S9B). Granzyme B and IFN-*γ* secreted by activated CD8^+^ T cells are known as the two key parameters that exert dominant roles in executing anticancer immune responses and inducing the apoptotic pathway [9, 52]. In this regard, the tumor-infiltrating IFN-*γ*^+^CD8^+^ activated T cells underwent similar trends of elevation after treatment with Eu-FBCP/PTX + aPL (14.9%), surpassing the treatment with Eu-s/PTX + aPL (12.4%), Eu-FBCP/PTX (6.52%), or aPL (10.5%) (Figs. 6i, S9C). Furthermore, Eu-FBCP/PTX + aPL significantly promoted the secretion of TNF-*α* in the tumor microenvironment compared with the treatment with Eu-s/PTX or aPL (Fig. S10A). The investigation of ratios between the tumor-infiltrating CD8^+^ T and Treg cells (reflecting tumor progression) revealed that Eu-FBCP/PTX + aPL can derive the highest ratio compared with other treatments (1.24-fold higher than Eu-s/PTX + aPL, 5.58-fold higher than Eu-FBCP/PTX, and 1.88-fold higher than free aPL) (Fig. S10B). The obtained results suggested that Eu-FBCP/PTX-induced ICD can be harnessed to assist stronger antitumor immune responses of aPL and trigger a great number of activated CD8^+^ T cells against MC-38 tumors.

### In Vivo Combinatorial Antitumor Effect

**Fig. 7:**
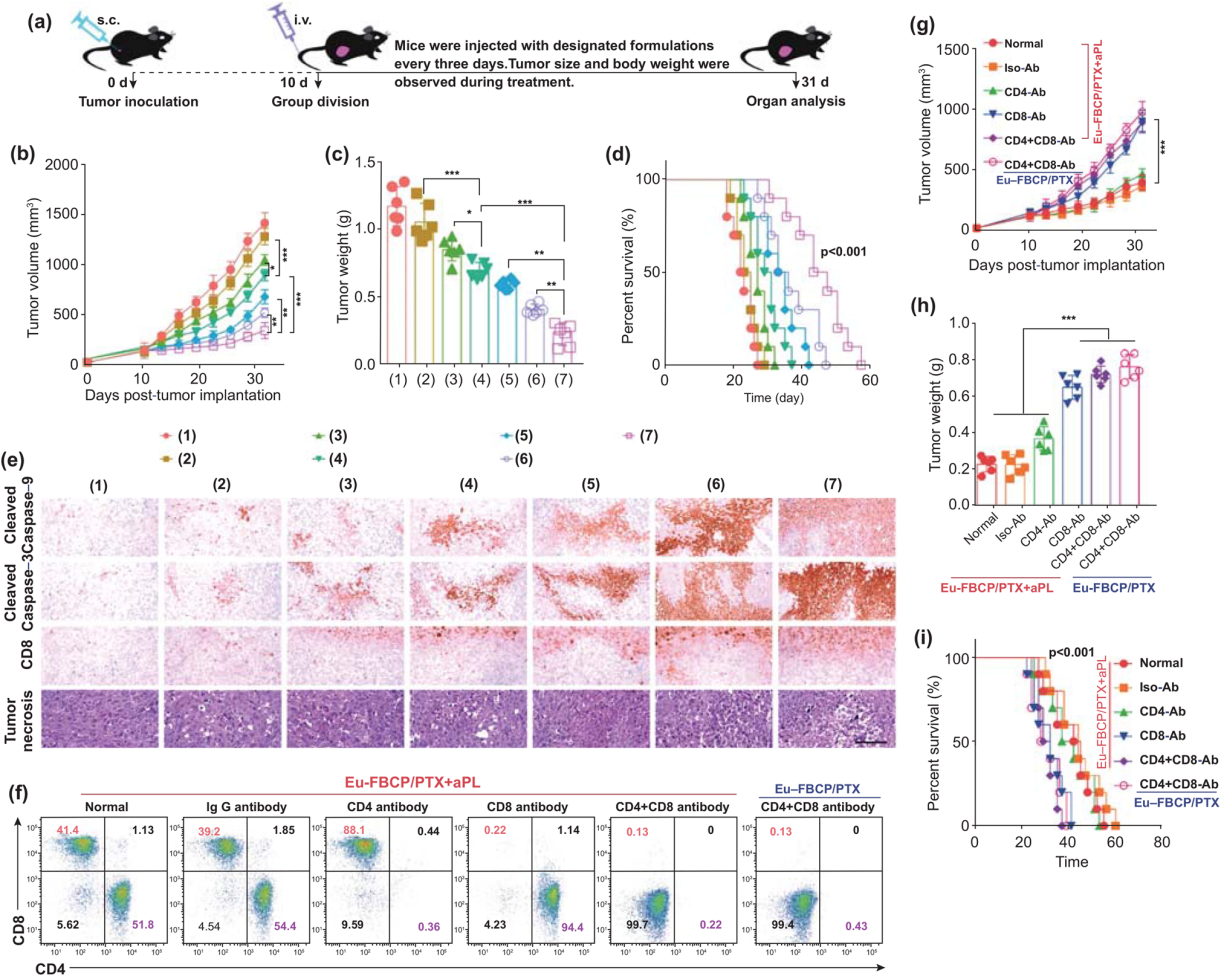
In vivo antitumor efficacies of Eu-FBCP/PTX and Eu-s/PTX in the absence (inducing chemical ICD) and presence (boosting immuno-antitumor activity) of aPL (*p < 0.05, **p < 0.01, and ***p < 0.001). **a** Schematic of the experimental schedules for in vivo antitumor studies with different treatments (1: PBS, 2: free PTX, 3: Eu-s/PTX, 4: Eu-FBCP/PTX, 5: aPL, 6: Eu-s/PTX + aPL, and 7: Eu-FBCP/PTX + aPL). **b**,** c** Time profiles of tumor sizes and final weights of tumors collected from the treated mice (1–7; six mice per group). **d** Survival curves of the treated mice (1–7; ten mice per group). **e** Histopathological and immunohistochemical expression of cleaved caspase-9, cleaved caspase-3, CD8^+^, and tumor necrosis factor in tumor sections obtained from the treated mice (1–7; six mice per group). **f** Immune cell generation after pretreatments with PBS (normal), IgG antibody (Iso-Ab), CD4 antibody (CD4-Ab), CD8 antibody (CD8-Ab), and CD8 + CD4 antibody (CD4 + CD8-Ab) to construct an immunocompromized MC-38 tumor-bearing mouse model. **g**,** h** Time profiles of tumor sizes and final weights of tumors collected from the immunocompromized mice singly treated with Eu-FBCP/PTX + aPL or Eu-FBCP/PTX (six mice per group). **i** Survival curves of the immunocompromized mice singly treated with Eu-FBCP/PTX + aPL or Eu-FBCP/PTX (ten mice per group)

Inspired by the superior activation of antitumor immune responses by combining Eu-FBCP/PTX with aPL, the therapeutic efficacy of these combinational treatments was evaluated in MC-38 tumor-bearing immunocompetent C57BL/6 mice. Tumors were allowed to grow to 80–120 mm^3^ in size before the group division and subsequent treatments (Fig. 7a). Eu-FBCP/PTX and Eu-s/PTX improved the efficacy of PTX and moderately suppressed the tumor growth in the absence of aPL, exhibiting 0.33- and 0.21-fold less than that from the treatment with free PTX (Fig. 7b). Eu-FBCP/PTX combined with aPL demonstrated dramatic suppression of tumor growth (0.62-fold less than Eu-FBCP/PTX and 0.51-fold less than free aPL), matching the combinational antitumor effects of Eu-FBCP/PTX + aPL in the previous bioassays. During the treatments, no notable impacts occurred in terms of mouse body weight, confirming the good biosafety of the treatments (Fig. S11). Digital images of the tumors and tumor weights were obtained on the last day and analyzed to elucidate the enhanced tumor suppression in mice from the treatment with Eu-FBCP/PTX + aPL (Figs. S12, 7c). Here, 0.66-fold and 0.55-fold lower tumor weights were observed upon the treatment with Eu-FBCP/PTX + aPL compared with those upon the treatments with Eu-FBCP/PTX and aPL, respectively. Correspondingly, Eu-FBCP/PTX + aPL exhibited the longest survival time, with a median of 41 days (cf*.* 29 days for Eu-FBCP/PTX and 33 days for aPL) (Fig. 7d). As shown in Fig. 7e, the examination of the tumor sections confirmed higher levels of apoptotic markers (cleaved caspase-9 and -3) for the treatments with Eu-FBCP/PTX and Eu-s/PTX than for free PTX, and the expression of these markers was further enhanced by combining with aPL. In particular, Eu-FBCP/PTX + aPL significantly promoted the CD8^+^ markers and exhibited more damaged areas in tumor sections compared with Eu-FBCP/PTX or free aPL, which may have been because of enhanced ICD triggered by Eu-FBCP/PTX. Analogous trends in the expression of HMGB1 and CRT in the tumor sections were observed (Fig. S13), and specifically, the enhanced ICD mediated by Eu-FBCP/PTX + aPL markedly downregulated the Ki-67 (tumor proliferation marker) and CD31 expression (angiogenesis marker), drastically abrogating tumor progression. According to the immunohistochemical indexes calculated from tumor sections, significant differences were also observed between Eu-FBCP/PTX + aPL and other treatments (Table S2). Hematoxylin and eosin (H&E) staining of major organs collected from the treated mice demonstrated no major pathological changes, supporting the biocompatibility of the nanosystem even combined with aPL (Fig. S14).

**Fig. 8:**
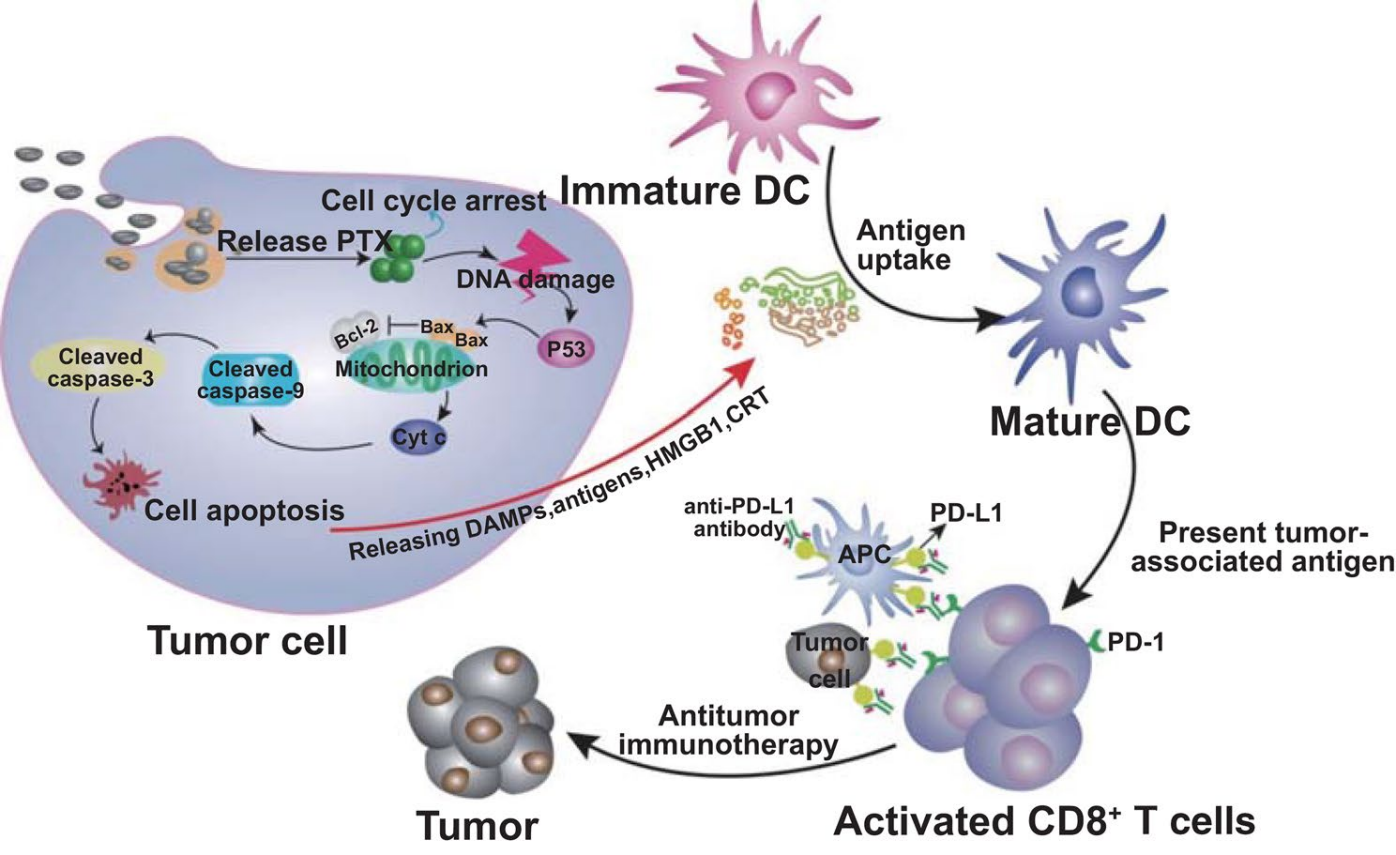
Schematic of a plausible model for the promoted efficacy from Eu-FBCP/PTX-induced ICD by incorporating Eu-FBCP/PTX with aPL. The Eu-FBCP/PTX entered the tumor cells through both phagocytosis and micropinocytosis for releasing PTX, inducing the G2/M phase in the cell cycle and intrinsic apoptosis. The apoptotic cancer cells significantly released HMGB1 and CRT that drove DC maturation and CD8^+^ T cell activation. This ICD induced by Eu-FBCP-PTX further facilitated the efficacy of aPL, which was greater than that from Eu-s/PTX because of the biomimetic property

The induction of ICD and the vital role of antitumor immune responses were further validated using MC-38 tumor-bearing CD4^−^, CD8^−^, and CD4^−^CD8^−^ immunocompromized C57BL/6 mice generated from antibody treatments. Compared with the percentages of CD4^+^ and CD8^+^ T cells in normal or IgG antibody-treated mice, the utilization of CD4, CD8, and CD4 + CD8 antibodies enabled the formation of CD4^−^, CD8^−^, or CD4^−^CD8^−^ immunocompromized mice (Fig. 7f). After the intravenous injection of Eu-FBCP/PTX + aPL, the tumor inhibition efficacy exhibited significant decreases in the CD8^−^ or CD4^−^CD8^−^ immunocompromized mice compared with that in the immunocompetent or IgG antibody-treated mice, exhibiting tumor growth profiles similar to those in the Eu-FBCP/PTX-treated CD4^−^CD8^−^ mice (Fig. 7g), whereas no significant losses in body weight were observed during the investigation period (Fig. S15). Correspondingly, digital images of the tumors and tumor weights harvested on the last day demonstrated significantly greater tumor sizes and heavier (almost 2.4-fold) tumor weights in the CD8^−^ or CD4^−^CD8^−^ immunocompromized mice than those in normal or IgG-treated mice (Figs. S16, 7h). In addition, in Eu-FBCP/PTX + aPL-treated CD8^−^ or CD4^−^CD8^−^ mice, the survival time was greatly reduced, with median survival of around 30 days, compared with 41 days in the normal or IgG-treated mice (Fig. 7i). These findings collectively revealed the dominant roles of Eu-FBCP/PTX-induced ICD and CD8^+^ T cells in enabling Eu-FBCP/PTX + aPL combinational treatment to act as antitumor chemo-immunotherapy (Fig. 8).

## Conclusions

An air–liquid two-phase electrospray designed to secure a balance between the mechanical (*CaRe*) and electrical (*B*_E_) parameters for stable bubble pressing of solutes inside drying droplets was shown to be feasible to continuously produce concave nanoparticles (< 300 nm in mean lateral dimension). This engineering approach was developed based on clinically relevant compounds (Eu and PTX) to provide biomimetic nanosystems (Eu-FBCP/PTX) for cancer chemo-immunotherapy (stronger ICD and antitumor immune response than those from Eu-s/PTX) without complex hydrothermal reactions and pre- and posttreatments, as well as undesirable and unexpected side effects from the use of unproven or newly developed nanomaterials. Because of the concave shape, the Eu-FBCP/PTX efficiently entered MC-38 tumor cells through both phagocytosis and micropinocytosis for enhancing selective PTX release to derive significant ICD from the promotion of DC maturation and CD8^+^ T cell activation. Specifically, the tumor cell apoptosis from Eu-FBCP/PTX treatment triggered the expression of HMGB1 and CRT, demonstrating greater DC maturation and activation of antitumor IFN-*γ*^+^CD8^+^ and granzyme B^+^CD8^+^ T cells compared with Eu-s/PTX. This significantly improved the efficacy of aPL (stimulating immune-antitumor environment) when Eu-FBCP/PTX was co-delivered with aPL (Eu-FBCP/PTX + aPL) for combination treatment, thus impeding tumor growth and the rapid death of tumor-bearing mice (effective for survival for about 60 days) by engineering only two clinically relevant compounds (even in the absence of release control agents). The strategy developed in this study not only provides a practical nanosystem only consisting of clinically relevant compounds (reducing R&D efforts and investment for clinical trials) to enhance the efficacy of cancer immunotherapeutics, but also offers a timely translatable method for the scalable manufacture of biomimetic nanosystems even utilizing currently available immune checkpoint blockade agents.

## Electronic supplementary material

Below is the link to the electronic supplementary material.

Supplementary file1 (PDF 2158 kb)
