# Supplementary material for: Artificial Nanoscale Erythrocytes from Clinically Relevant Compounds for Enhancing Cancer Immunotherapy
Source: Nanomicro Lett. 2020 Apr 13;12:90. doi: 10.1007/s40820-020-00428-y (PMC7770689; doi:10.1007/s40820-020-00428-y)
Supplement: Supplementary file 1 — Supplementary file1 (PDF 2158 kb) [file 40820_2020_428_MOESM1_ESM.pdf]

Supporting Information for

Artificial Nanoscale Erythrocytes from Clinically Relevant Compounds for Enhancing Cancer Immunotherapy

Wenquan Ou[1], Kang Sik Nam[2], Dae Hoon Park[2], Jungho Hwang[2, *], Sae Kwang Ku[3], Chul Soon Yong[1], Jong Oh Kim[1, *], Jeong Hoon Byeon[4, *]

[1]College of Pharmacy, Yeungnam University, Gyeongsan 38541, Republic of Korea

[2]School of Mechanical Engineering, Yonsei University, Seoul 03722, Republic of Korea

[3]College of Korean Medicine, Daegu Haany University, Gyeongsan 38610, Republic of Korea

[4]School of Mechanical Engineering, Yeungnam University, Gyeongsan 38541, Republic of Korea

Wenquan Ou and Kang Sik Nam contributed equally to this work

*Corresponding authors. E-mail: hwangjh@yonsei.ac.kr (Jungho Hwang); jongohkim@yu.ac.kr (Jong Oh Kim); postjb@yu.ac.kr (Jeong Hoon Byeon)

Supplementary Figures and Tables

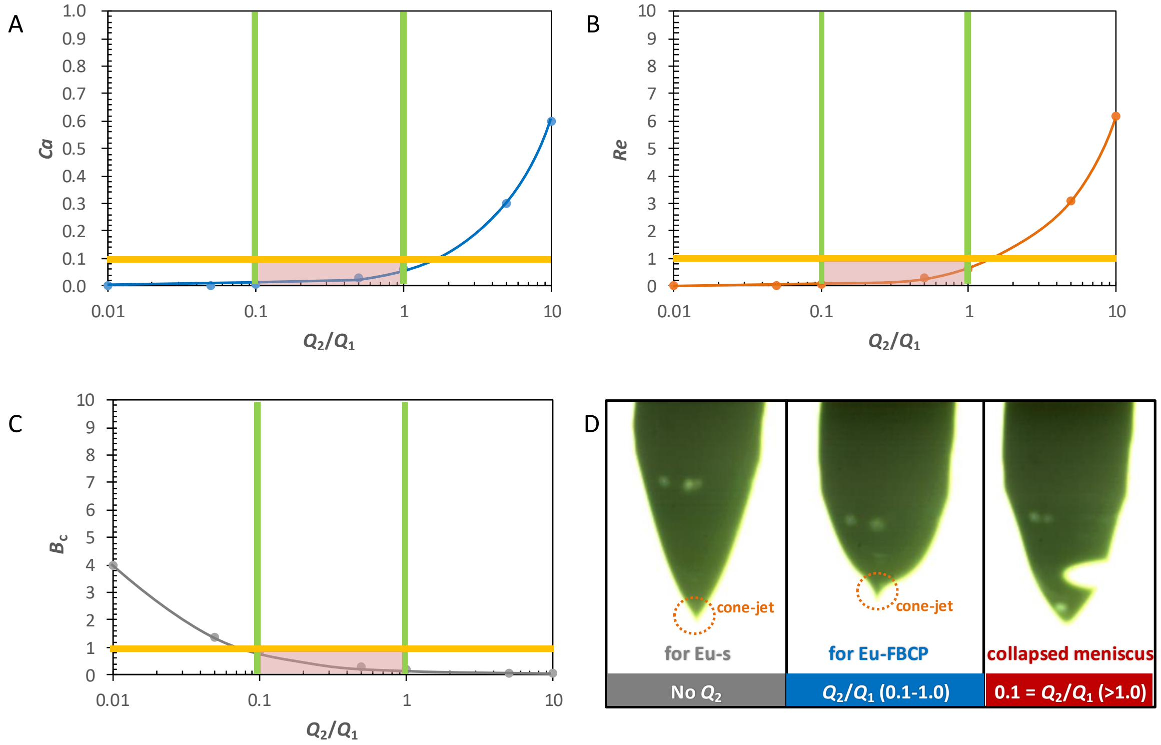

Fig. S1 Mechanical and electrical parameters with high-speed camera images of the two-phase electrospray. (**A, B**) Capillary (*Ca*) and Reynolds (*Re*) numbers as a function of volumetric flow ratio between the Eu/PTX solution (Q_1) and air (Q_2). (**C**) Characteristic balance (B_c) between the B_E^2 and *CaRe* exhibiting a balance between the electrical and mechanical parameters of the electrospray. (**D**) High-speed camera images of meniscus shapes from different Q_2/Q_1 operations

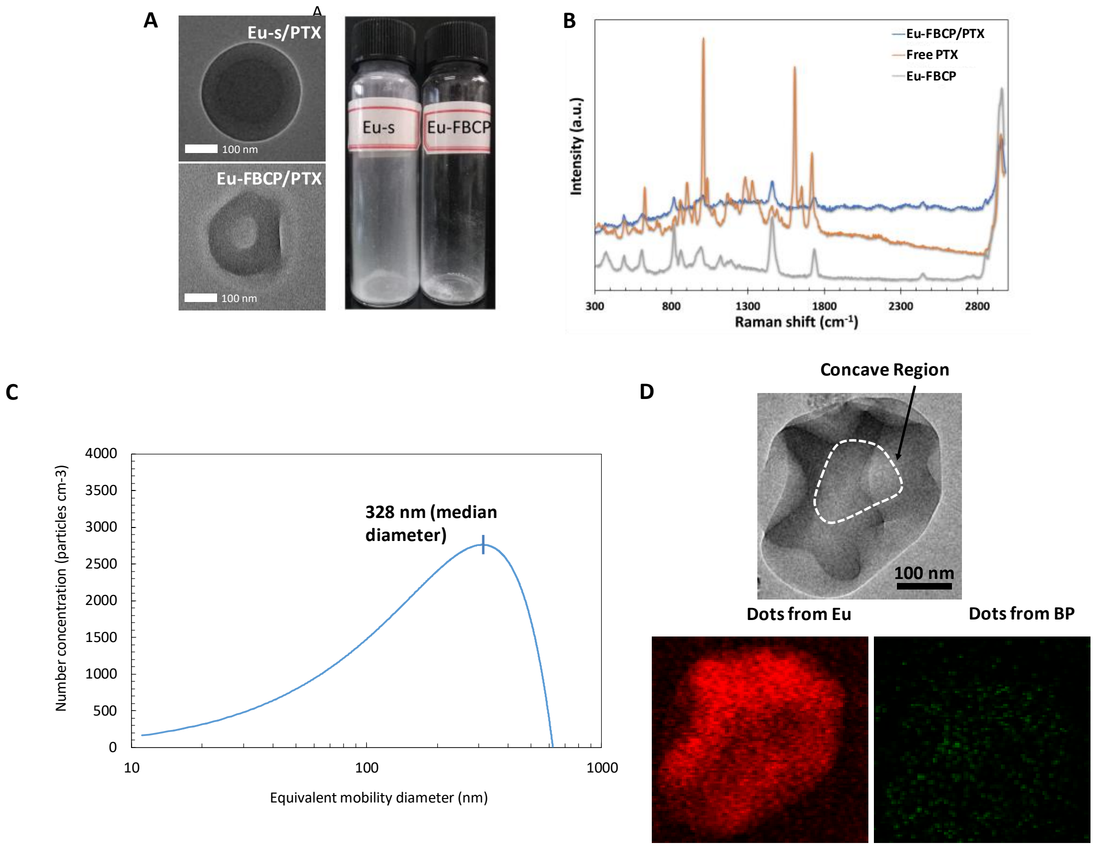

Fig. S2 (**A**) High-resolution TEM and digital images of collected powder forms of Eu-s/PTX (from single-phase electrospray) and Eu-FBCP/PTX (from two-phase electrospray). The TEM images exhibit clear difference in core region contrast between Eu-s/PTX (thick core) and Eu-FBCP/PTX (thin core). (**B**) Raman spectra of Eu-FBCP/PTX, as well as individual PTX and Eu-FBCP. (**C**) In-flight size distribution of Eu-FBCP/PTX observed using a SMPS by direct vacuum sampling of floating particles right after electrospraying. (D) A representative TEM image and its energy-dispersive X-ray maps (red: carbon dots, green: phosphorus dots) from electrospray of BP NP included Eu/PTX solution

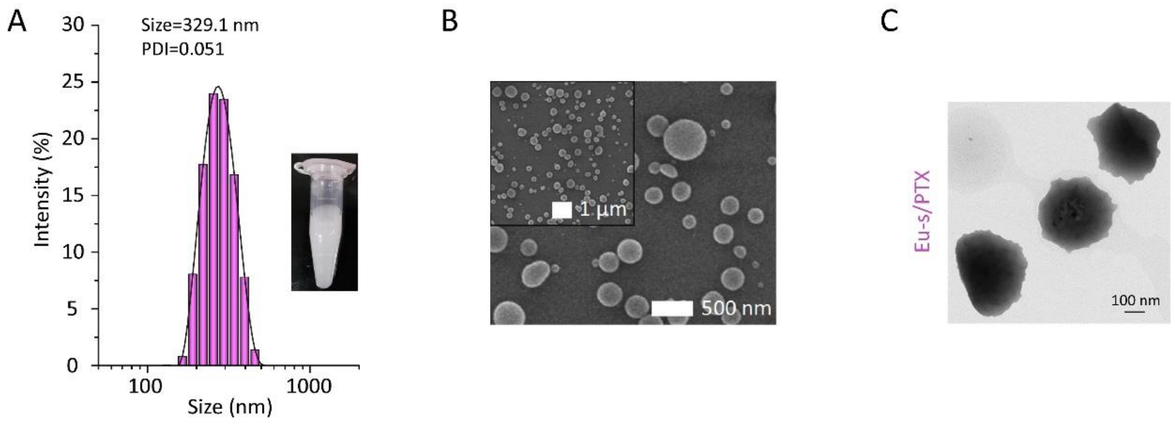

Fig. S3 Physical characterization of Eu-s/PTX for comparison. (**A**) DSL size distribution of Eu-s/PTX dispersed in PBS (refer to the inset digital image). (**B, C**) SEM (low- and high-magnification) and TEM images of Eu-s/PTX collected on a carbon-coated copper grid

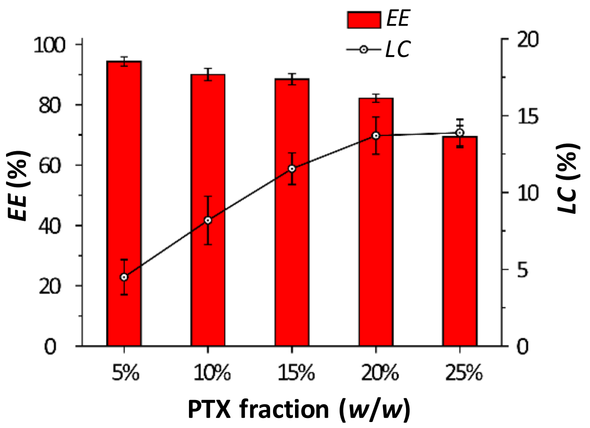

Fig. S4 Percentage *EE* and *LC* profiles of Eu-FBCP/PTX with increasing PTX content from 5% to 25% (PTX-to-Eu weight ratio) (n = 6)

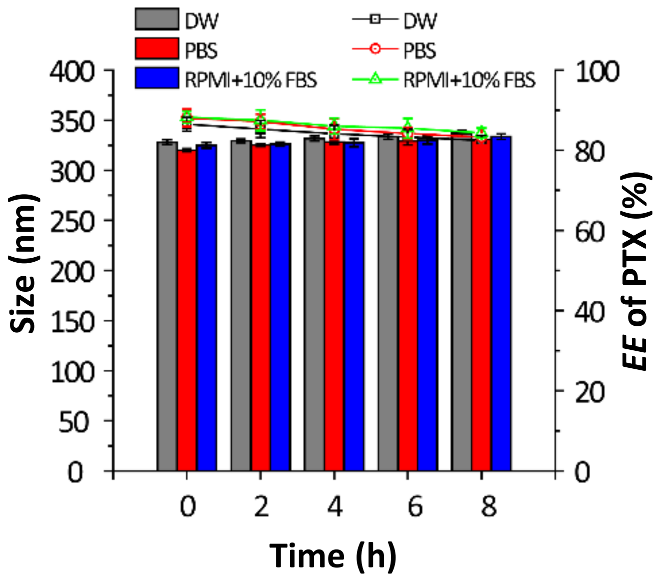

Fig. S5 Time profiles of mean DLS size and *EE* of Eu-FBCP/PTX dispersed in DW, PBS, or RPMI + 10% FBS for 8 h (n = 6)

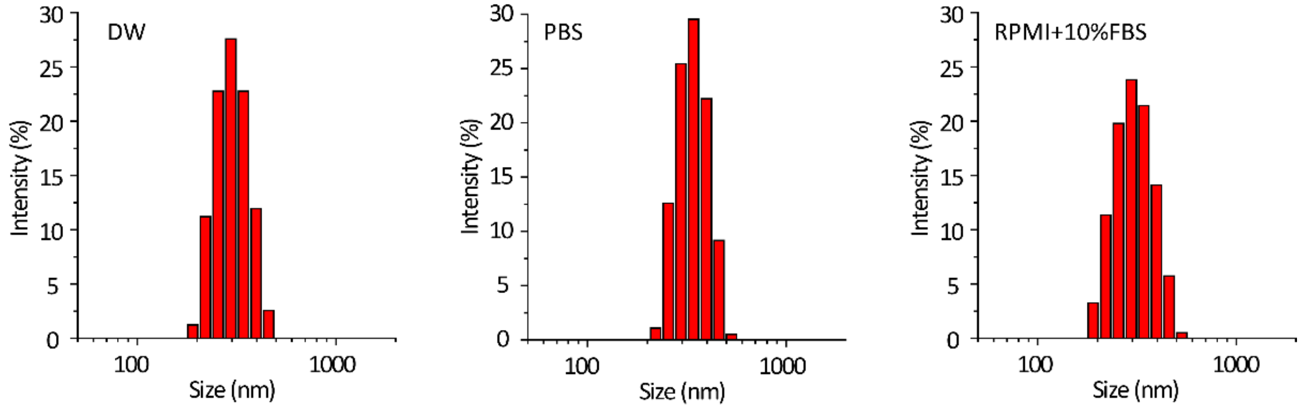

Fig. S6 DLS size distributions of Eu-FBCP/PTX after 8 h dispersed in DW, PBS, and RPMI + 10% FBS

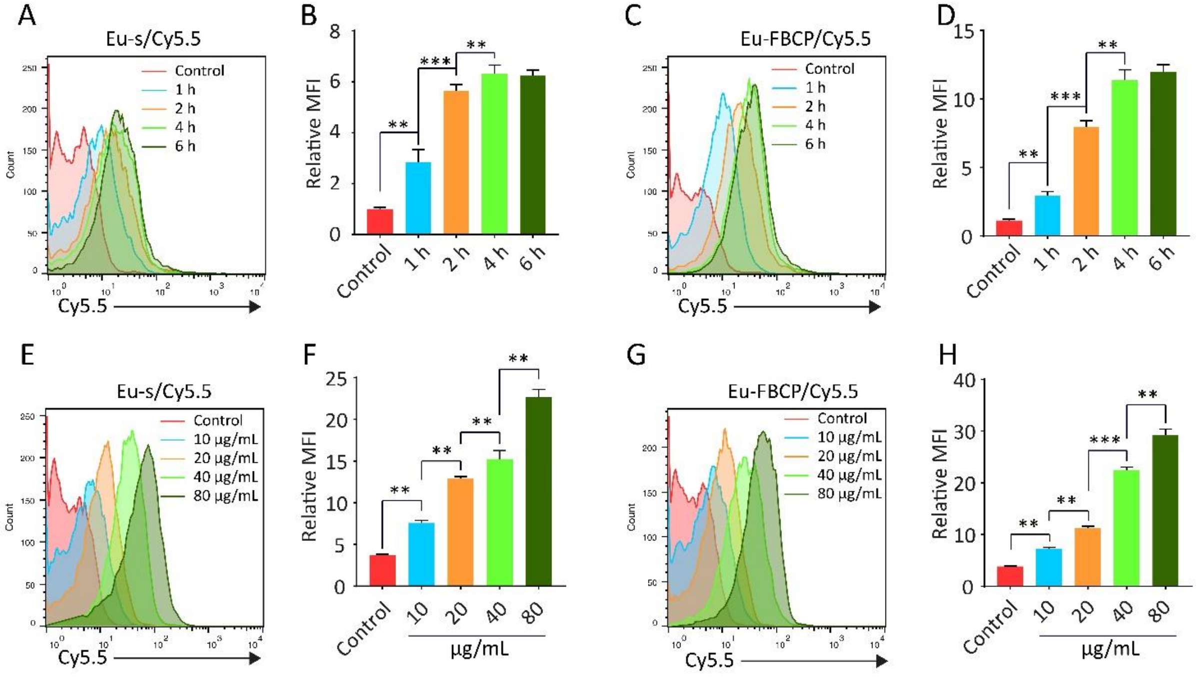

Fig. S7 FACS analyses to examine time- and dose-dependent cellular uptake of Eu-FBCP, as well as Eu-s for comparison. (**A–D**) Time-dependent uptake profiles and corresponding quantified indices of Cy5.5 tagged on Eu-FBCP and Eu-s under an identical dose (n = 3). (**E–H)** Dose-dependent uptake profiles and corresponding quantified indices of Cy5.5 tagged on Eu-FBCP and Eu-s under an identical incubation time (n = 3; $^{**}p < 0.01$ and $^{***}p < 0.001$)

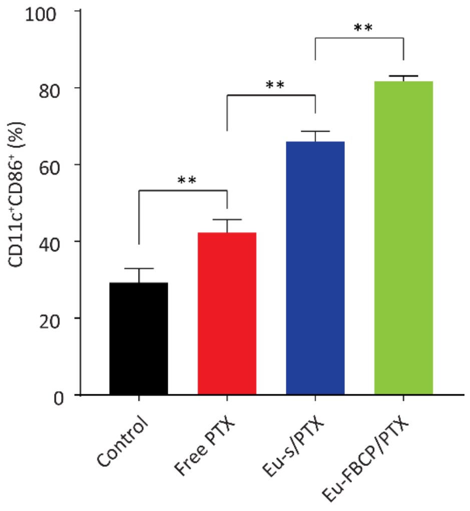

Fig. S8 Mature DCs exposed to tumor-associated antigen generated from MC-38 cells treated with Eu-FBCP/PTX, as well as free PTX and Eu-s/PTX for comparison. CD11c$^+$CD86$^+$ cells are the indicators of mature DCs (n = 3; $^{**}p < 0.01$).

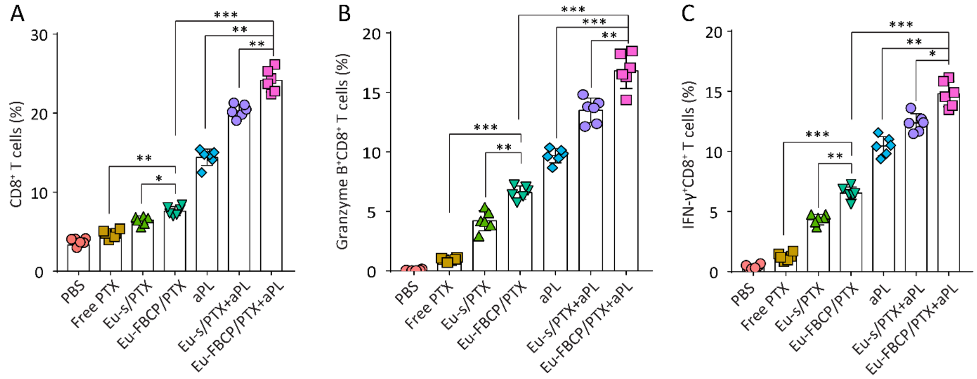

Fig. S9 (**A**) Infiltrating CD8$^+$ T cells in tumor microenvironment after treatments with Eu-FBCP/PTX and Eu-FBCP/PTX + aPL, as well as PBS, free PTX, aPL, Eu-s/PTX, and Eu-s/PTX + aPL for comparison (n = 6). (**B, C**) Intratumoral granzyme B$^+$ and IFN-γ^+CD8$^+$ T cells after the treatments (n = 6; $^*p < 0.05$, $^{**}p < 0.01$, and $^{***}p < 0.001$)

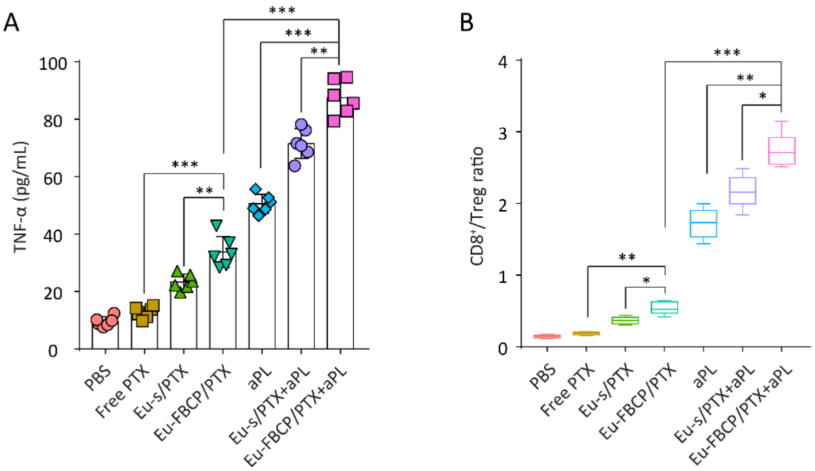

Fig. S10 (**A**) TNF-α levels in tumor microenvironment from mice treated with Eu-FBCP/PTX and Eu-FBCP/PTX + aPL, as well as PBS, free PTX, aPL, Eu-s/PTX, and Eu-s/PTX + aPL for comparison (n = 6). (**B**) Infiltrating CD8$^+$ T-to-Treg cell ratios in tumors isolated from the treated mice (n = 6; $^*p < 0.05$, $^{**}p < 0.01$, and $^{***}p < 0.001$)

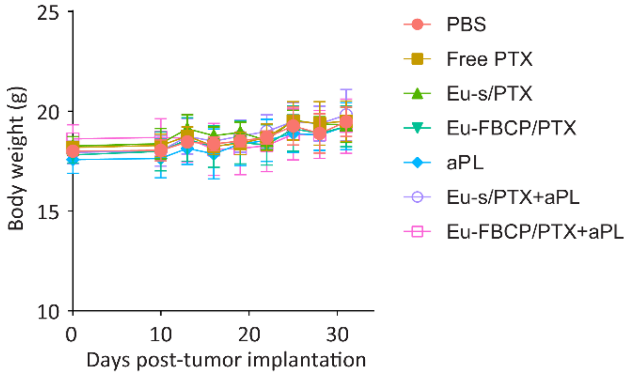

Fig. S11 Body weight profiles of MC-38 tumor-bearing mice treated with Eu-FBCP/PTX and Eu-FBCP/PTX + aPL, as well as PBS, free PTX, aPL, Eu-s/PTX, and Eu-s/PTX + aPL for comparison (n = 6)

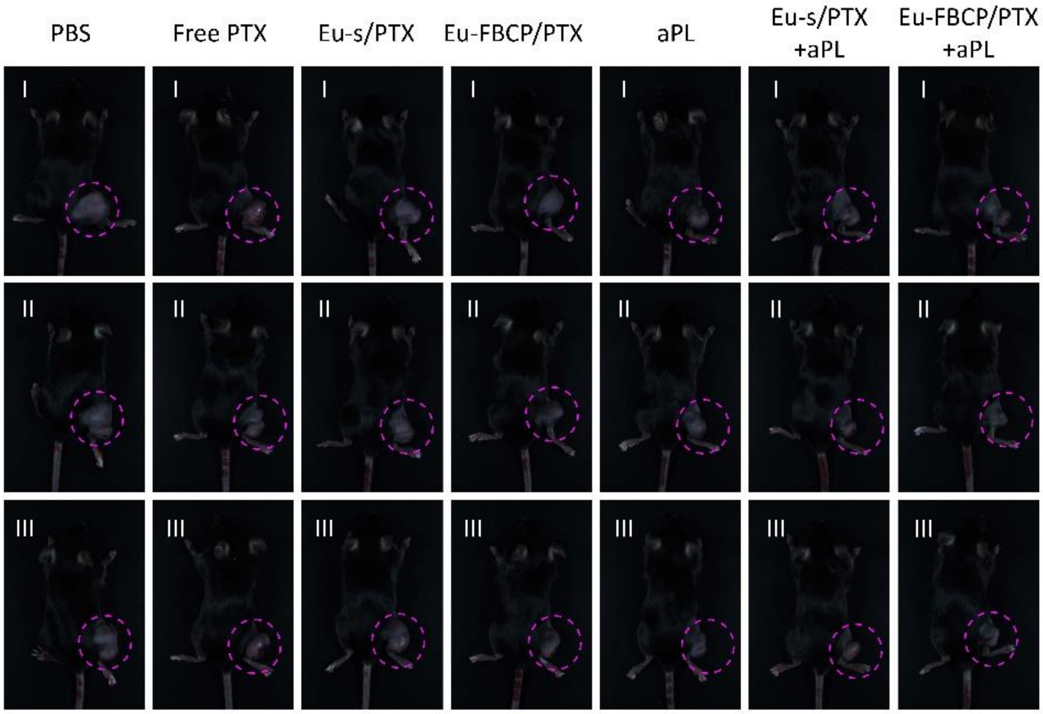

Fig. S12 Digital images of mice taken at the final day after treatments with Eu-FBCP/PTX and Eu-FBCP/PTX + aPL, as well as PBS, free PTX, aPL, Eu-s/PTX, and Eu-s/PTX + aPL for comparison

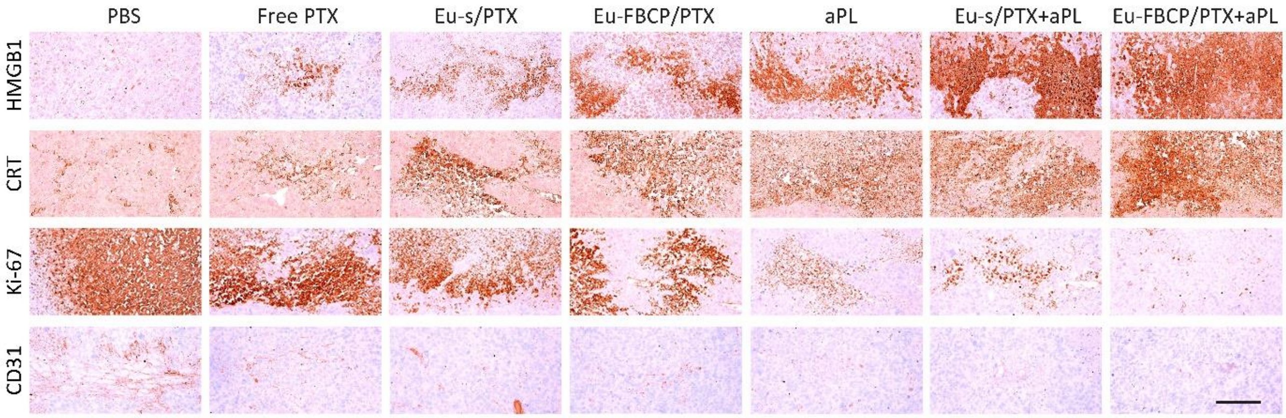

Fig. S13 Histopathological and immunohistochemical images to assess levels of HMGB1, CRT, Ki-67, and CD31 in tumors obtained from MC-38 tumor-bearing mice treated with Eu-FBCP/PTX and Eu-FBCP/PTX + aPL, as well as PBS, free PTX, aPL, Eu-s/PTX, and Eu-s/PTX + aPL for comparison

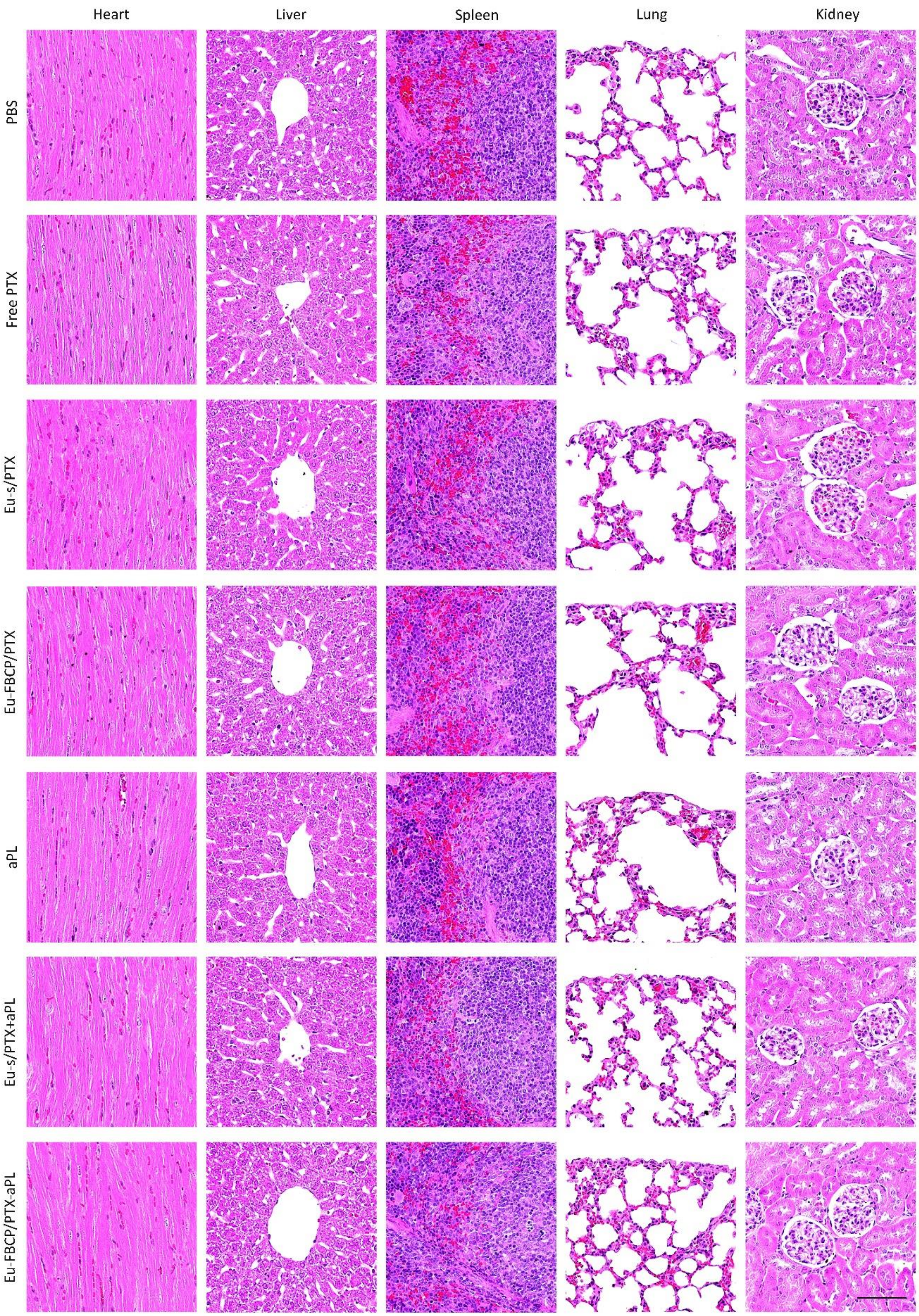

Fig. S14 *In vivo* histopathological images for hearts, livers, spleens, lungs, and kidneys isolated from mice treated with Eu-FBCP/PTX and Eu-FBCP/PTX + aPL, as well as PBS, free PTX, aPL, Eu-s/PTX, and Eu-s/PTX + aPL for comparison

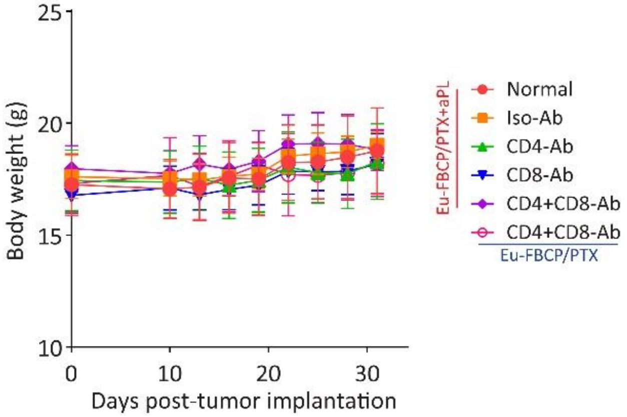

Fig. S15 Body weight profiles of differently immunocompromized mice during treatments with Eu-FBCP/PTX with and without aPL

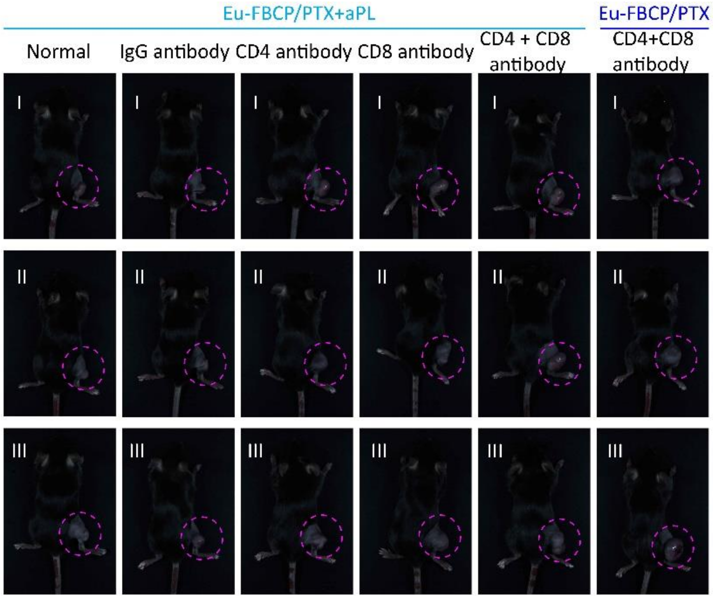

Fig. S16 Digital images of differently immunocompromized mice taken at the final day after treatments with Eu-FBCP/PTX with and without aPL

Table S1 Summary of DSL size distributions of Cy5.5-labeled Eu (RL or RS)-FBCP and Eu (RL or RS)-s

Groups	Size (nm)	PDI
Eu-s/Cy5.5 (RL)	318.7 ± 2.5	0.109 ± 0.014
Eu-FBCP/Cy5.5 (RL)	321.3 ± 1.8	0.094 ± 0.008
Eu-s/Cy5.5 (RS)	322.2 ± 3.1	0.142 ± 0.017
Eu-FBCP/Cy5.5 (RS)	321.4 ± 2.6	0.111 ± 0.036

Table S2 Histomorphometrical analysis of tumor masses, taken from MC-38 allograft tumor-bearing mice treated with Eu-FBCP/PTX and Eu-FBCP/PTX + aPL, as well as PBS, free PTX, aPL, Eu-s/PTX, and Eu-s/PTX + aPL for comparison

Groups<br>Items	G1<br>(PBS control)	Test formula treated groups					
		G2	G3	G4	G5	G6	G7
TCV (%/mm²)	87.57±4.88	74.12±3.92[a]	62.86±3.55[ab]	50.74±5.22[abd]	39.53±5.17[abde]	29.42±4.36[abdef]	15.74±4.88[abdefg]
Immunoreactive cell percentages (%/mm² of tumor mass)							
Cleaved-Caspase-3	4.83±1.95	14.84±3.42[a]	25.43±3.51[ab]	39.66±3.72[abd]	48.79±3.60[abde]	64.25±4.02[abdef]	78.79±3.49[abdefg]
Cleaved-Caspase-9	6.93±2.52	15.65±3.10[a]	21.40±2.83[ac]	37.25±3.40[abd]	45.24±4.81[abde]	62.95±3.77[abdef]	76.62±4.79[abdefg]
Ki-67	53.12±6.45	40.18±4.02[h]	32.03±3.47[hj]	23.03±3.09[hik]	14.17±2.97[hikl]	8.33±1.44[hiklm]	3.30±1.36[hiklmn]
CD31	47.65±2.96	36.39±3.14[a]	29.64±2.83[ab]	23.35±2.35[abd]	18.99±2.36[abde]	9.66±2.11[abdef]	3.58±1.30[abdefg]
HMGB1	2.64±1.17	12.80±2.72[a]	23.55±3.27[ab]	37.36±4.83[abd]	49.09±4.17[abde]	63.27±6.44[abdef]	81.77±6.29[abdefg]
CRT	6.38±2.64	17.45±2.48[a]	28.62±3.90[ab]	41.11±2.81[abd]	53.81±5.44[abde]	64.62±5.23[abdef]	83.18±7.24[abdefg]
Immunoreactive cell numbers (cells/mm² of tumor mass)							
CD8	46.33±10.31	139.33±29.98[h]	279.00±19.30[hi]	378.67±24.68[hik]	624.33±90.77[hikl]	1419.83±310.12[hiklm]	2636.33±596.09[hiklmn]

Values are expressed as mean ± SD of six tumor mass histological fields.

Groups: G1 = PBS; G2 = free PTX; G3 = Eu-s/PTX; G4 = Eu-FBCP/PTX; G5 = aPL; G6 = Eu-s/PTX + aPL; and G7 = Eu-FBCP/PTX + aPL.

[a]$p < 0.01$ as compared to G1 by Fisher's least significant difference (LSD) test;

[b]$p < 0.01$ and [c]$p < 0.05$ as compared to G2 by LSD test;

[d]$p < 0.01$ as compared to G3 by LSD test;

[e]$p < 0.01$ as compared to G4 by LSD test;

[f]$p < 0.01$ as compared to G5 by LSD test;

[g]$p < 0.01$ as compared to G6 by LSD test;

[h]$p < 0.01$ as compared to G1 by Mann–Whitney (MW) test;

[i]$p < 0.01$ and [j]$p < 0.05$ as compared to G2 by MW test;

[k]$p < 0.01$ as compared to G3 by MW test;

[l]$p < 0.01$ as compared to G4 by MW test;

[m]$p < 0.01$ as compared to G5 by MW test; and

[n]$p < 0.01$ as compared to G6 by MW test.
